# Genomic mosaicism in paternal sperm and multiple parental tissues in a Dravet syndrome cohort

**DOI:** 10.1038/s41598-017-15814-7

**Published:** 2017-11-15

**Authors:** Xiaoxu Yang, Aijie Liu, Xiaojing Xu, Xiaoling Yang, Qi Zeng, Adam Yongxin Ye, Zhe Yu, Sheng Wang, August Yue Huang, Xiru Wu, Qixi Wu, Liping Wei, Yuehua Zhang

**Affiliations:** 1 Center for Bioinformatics, State Key Laboratory of Protein and Plant Gene Research, School of Life Sciences, Peking University, Beijing, 100871 China; 2 Department of Pediatrics, Peking University First Hospital, Beijing, 100034 China; 3 Peking-Tsinghua Center for Life Sciences, Beijing, 100871 China; 4 Academy for Advanced Interdisciplinary Studies, Peking University, Beijing, 100871 China; 5 School of Life Sciences, Peking University, Beijing, 100871 China; 6 National Institute of Biological Sciences, Beijing, 102206 China; 7 College of Biological Sciences, China Agricultural University, Beijing, 100094 China; 8 Present Address: Room 342, School of Life Sciences, Wang Ke-Zhen Building, 5th Yiheyuan Road, Peking University, Beijing, 100871 China; 9 Present Address: Room 307, Center for Bioinformatics, Wang Ke-Zhen Building, 5th Yiheyuan Road, Peking University, Beijing, 100871 China; 10 Present Address: Department of Pediatrics, Peking University First Hospital, 8th Xi’anmen Avenue, Beijing, 100034 China

## Abstract

Genomic mosaicism in parental gametes and peripheral tissues is an important consideration for genetic counseling. We studied a Chinese cohort affected by a severe epileptic disorder, Dravet syndrome (DS). There were 56 fathers who donated semen and 15 parents who donated multiple peripheral tissue samples. We used an ultra-sensitive quantification method, micro-droplet digital PCR (mDDPCR), to detect parental mosaicism of the proband’s pathogenic mutation in *SCN1A*, the causal gene of DS in 112 families. Ten of the 56 paternal sperm samples were found to exhibit mosaicism of the proband’s mutations, with mutant allelic fractions (MAFs) ranging from 0.03% to 39.04%. MAFs in the mosaic fathers’ sperm were significantly higher than those in their blood (*p* = 0.00098), even after conditional probability correction (*p’* = 0.033). In three mosaic fathers, ultra-low fractions of mosaicism (MAF < 1%) were detected in the sperm samples. In 44 of 45 cases, mosaicism was also observed in other parental peripheral tissues. Hierarchical clustering showed that MAFs measured in the paternal sperm, hair follicles and urine samples were clustered closest together. Milder epileptic phenotypes were more likely to be observed in mosaic parents (*p* = 3.006e-06). Our study provides new insights for genetic counseling.

## Introduction

Sporadic cases of parental pathogenic mosaic mutations have been documented in more than 100 Mendelian disorders^1–5^, such as Charcot-Marie-Tooth disease^6^, Dravet syndrome^7^, Freeman-Sheldon syndrome^8^, and epilepsy in females with mental retardation^9^. Parental germline mosaicism and somatic mosaicism in patients have been studied in families affected by Alport syndrome^10^, focal cortical dysplasia type II^11^, extracranial arteriovenous malformation^12^, and epilepsy-related neurodevelopmental disorders^13^ at the cohort level. Parental mosaicism has also been reported in cohorts of complex neurological and psychiatric disorders, such as autism spectrum disorder^14–17^, intellectual disability^18^, and epileptic encephalopathies^19^. In these reported families, parents with mosaic mutations are either normal^20^ or have milder clinical phenotypes^15^ compared to their affected children^21^. However, paternal sperm samples have not been studied in monogenic epileptic disorders at the cohort level.

Paternal sperm samples can be obtained non-invasively, and they provide useful genetic information. Studies that have focused on germline mosaicism have shown increased paternal mutation rates^22^, and large scale *de novo* mutation data show significant changes in the proportions of mutant alleles in sperm as paternal age increases^23^. The spermatogonial selfish selection mechanism theory was previously proposed to explain this phenomenon in non-cancer genetic disorders caused by cancer-related genes^24–28^. However, recent population genetic models have suggested that shared genetic risk factors might be an alternative explanation for the elevated risks of psychiatric disorders^29^. The postzygotic single nucleotide mosaic mutation profile between paternal sperm and blood samples remains largely unknown at the cohort level for monogenic epileptic disorders caused by non-cancer genes.

There have been extensive studies on genomic differences between tissues, revealing somatic aneuploidy, copy number variations (CNVs)^30^, and transposable elements^31^ in various fetal tissues such as brain^32,33^, skin^32^, extraembryonic cells^33–35^, ovarian cells, and postnatal tissues^36,37^ such as blood, brain, skin, liver, and germline cells^38,39^. The differences can occur at the level of tissues or single cells. Brain-specific mosaic mutations have been successfully identified and validated, and were reported to be responsible for the phenotypes of the mutation carriers^11,37,40^. Mutations specifically identified in male germline cells have shown differences in mutation rate^41^ that are thought to be shaped by selective pressure^22,42^. However, the differences in mutation frequencies of postzygotic single nucleotide mosaicism are not yet well understood in the context of multiple tissues or in sperm cells at the cohort level for neurological disorders. The existing studies using multiple samples for disorders caused by cancer genes, such as *COLA5* ^10^, *MAP2K1* ^12^, and *ASXL1* ^36^ were limited by the detection methods because next-generation sequencing (NGS) approaches and traditional digital PCR based methods have a detection and quantification limit of 0.5–1%. Studies using multiple samples for disorders caused by non-cancer genes, such as *ATP1A3* ^35^, *MEFV* ^38^, *PCDH19* ^39,40^, *SCN1A* ^21^, and *SCN5A* ^42^, were limited by their sample sizes, because the collection of a large cohort is difficult and reports tend to appear as case studies.

The severe epileptic syndrome Dravet syndrome (DS, MIM: 607208), which was previously described as severe myoclonic epilepsy of infancy (SMEI), often occurs in infants under 12 months of age^43^. The main phenotype of DS is the occurrence of multiple seizure types that are fever-sensitive and refractory. The syndrome also involves psychomotor developmental delay after seizure onset^44,45^. Of DS probands, 70–80% were found to carry deleterious mutations in the gene encoding the alpha subunit of the sodium channel neuronal type I (*SCN1A*, HGNC: 10585, MIM: 182389)^44,46^. We previously identified parental mosaicism for approximately 10% of seemingly “*de novo*” *SCN1A* mutations using PGM amplicon sequencing for mosaicism (PASM)^21^, which can detect mutations with mutant allelic fractions (MAFs) of 0.5%. However, we need a more accurate approach to distinguish the differences of MAFs between tissues. The next-generation digital PCR technology, Raindrop micro-droplet digital PCR (mDDPCR) offers an ultra-sensitive and cost-effective alternative; it can generate up to 10 million droplets in an emulsion system^47–49^ and can theoretically detect mutations with MAFs of 10^−4^ or lower^50–53^. In this study, we used mDDPCR on a selected Chinese cohort consisting of 112 families out of a set of 719 families affected by DS. Of these, 56 fathers donated sperm samples, and 15 parents donated multiple peripheral tissue samples. We detected parental mosaicism of the proband’s pathogenic mutation in *SCN1A*. The mosaic statuses were also quantified by PASM. We examined differences in the postzygotic mutation patterns between paternal sperm and parental tissue samples, and we summarized the phenotype-genotype correlations between different groups of parents and the mosaic probands.

## Results

### Parental mosaicism in blood samples from *SCN1A* mutated DS families

**Figure 1:**
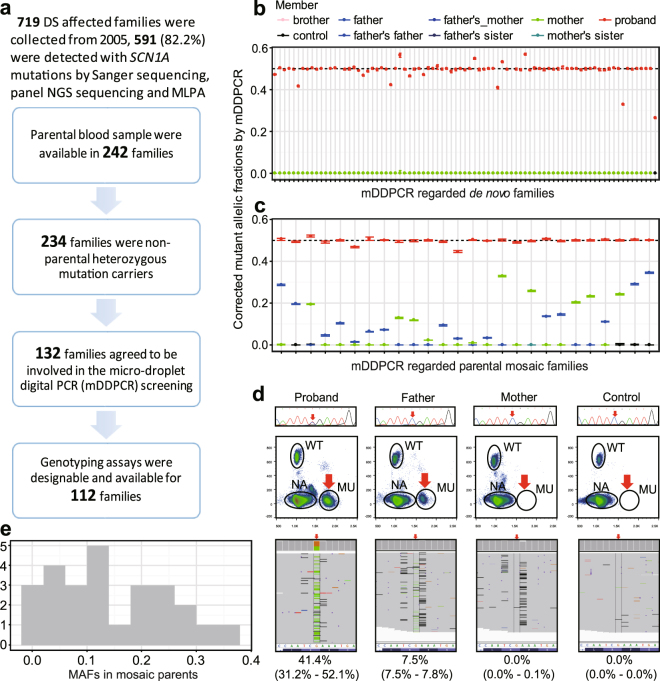
DS cohort analyzed in the study and mDDPCR results identifying parental mosaicism in blood samples. (**a**) Description of the cohort analyzed in this study. A total of 719 DS affected families identified since 2005 were included. Sanger sequencing, panel NGS sequencing and MLPA detected *SCN1A* mutations from probands in 591 families. Peripheral blood samples from both parents are available for 242 of the families, and there are 8 probands among these families that inherited mutations from their parents according to the Sanger screening results. A total of 132 of the families provided blood samples and agreed to be included in the mDDPCR screening. TaqMan genotyping assays were able to be conducted for 112 families. (**b**) Overview of mDDPCR results that identified “*de novo*” mutations in families. The y-axis shows the maximum likelihood estimates of MAFs, and the error bars show the 95% binomial CIs calculated from the mDDPCR results. The probands have corrected MAFs between 40% and 60%, whereas the allele frequencies detected in the parents and negative controls were all under the detection limit. (**c**) Overview of MAFs measured in candidate parental mosaic families. Blood samples from family members are plotted. For each parental mosaic family, only one mosaic parent had an MAF 95% binomial CI between 50% and 0%. Blood samples from the non-mosaic parent and the negative control show MAFs of approximately 0%. (**d**) A representative result for parental mosaicism identified in family DS314 is shown. The PCR Sanger sequencing chromatogram, mDDPCR flow cytometry scatter plots and PASM raw IGV views and CI calculations after Bayesian modeling for the blood samples from the DS314 family are provided. Detailed mDDPCR flow cytometry scatter plots for all parental mosaic families are provided in Supplementary Fig. S7. (**e**) Histogram of the MAF distribution for parental blood samples from parental mosaic families.

Of the 719 patients in the cohort from Peking University First Hospital, 591 (82%) have been found to carry *SCN1A* mutations based on Sanger sequencing. Blood samples from the parents were available for this study from 242 families, 234 of which did not have parents carrying *SCN1A* mutations, and 132 of these families agreed to enroll in this study of mosaicism. Thirty-three (25.0%) of the *SCN1A* mutations have never been reported (Supplementary Table S1), and their probabilities of deleterious effects were predicted to be similar to those of the reported *SCN1A* mutations causing DS (Supplementary Fig. S1). mDDPCR was carried out for 112 families for which TaqMan assays were available (Fig. 1a and Methods). For 56 of these families, the fathers donated semen samples, and 15 parents donated samples from multiple peripheral tissues, including saliva, urine, hair follicles and oral epithelium.

mDDPCR was carried out to detect mosaicism. The pipeline for mDDPCR is depicted in Supplementary Fig. S2 and described in the Methods section. An end-point genotyping qPCR analysis was first carried out for genotyping assays (Supplementary Fig. S3). The detection limit of mDDPCR determined by using a sequential dilution benchmarking test (Supplementary Fig. S4). Mutations detected with the lower bound of the 95% binominal confidence interval with an MAF higher than 0.01% were selected as positive mosaic cases.According to the mDDPCR results, after correction of the MAF based on considering homologous sequences, all probands examined had MAFs between 40% and 60% (Fig. 1b,1c, Supplementary Fig. S5, and Supplementary Fig. S6), except for two putative mosaic probands (DS315 with an MAF of 32.98% and DS330 with an MAF of 26.48%, Fig. 1b). Parental mosaicism in the blood was found in 26 families (Fig. 1c, Supplementary Fig. S7, Supplementary Fig. S8 and Table 1). In each mosaic family, only one of the parents was genotyped as mosaic by mDDPCR, whereas no detectable MAFs were observed in the other parent. Figure 1d shows the DS314 family as an example: in flow cytometry scatter plots, a signal cluster demonstrating the mutant alleles (MU) was detected in a similar position as in the blood samples from the father and the proband, whereas MU clusters were not observed in the blood samples from the non-mosaic parent and the negative controls (Fig. 1d and Supplementary Fig. S7). Parental mosaicism was further validated by PASM. Sanger sequencing results of the families were also provided (Fig. 1d and Table 1).

**Table 1:** Locations and MAFs of the validated parental mosaic mutations in blood, sperm and other peripheral tissue samples.

Family ID	Parental Origin	Sanger Screening	Variant information					Proband’s blood		Control’s blood		Father’s blood		Mother’s blood		Father’s	Mosaic parent’s other peripheral tissue samples			
			Chrom-osome	Position^a^	Ref	Alt	Exon	mDDPCR^b^	PASM	mDD-PCR	PASM	mDD-PCR	PASM	mDD-PCR	PASM	Sperm/semen	Oral epithelium	Saliva	Hair follicle	Urine
DS001	Paternal	Detectable	2	166904194	T	—	Exon8	50.39%	49.20%	0.00%	0.80%	28.59%	32.60%	0.00%	5.60%	39.04%	NA^d^	21.44%	34.43%	35.89%
DS003	Paternal	Detectable	2	166894639	C	T	Exon15	49.29%	46.10%	0.01%	0.12%	19.43%	18.21%	0.01%	ND^c^	23.19%	11.19%	19.70%	11.93%	14.16%
DS017	paternal	Undetectable	2	166848438	G	A	Exon26	49.19%(16.40%)	52.90%	0.00%(0.00%)	0.00%	4.36%(1.45%)	4.00%	0.01%(0.00%)	0.00%	7.52%(2.51%)	NA	2.58%(0.86%)	3.85%(1.28%)	NA
DS101	paternal	Undetectable	2	166848230	T	C	Exon26	50.05%	46.17%	0.00%	0.10%	6.31%	6.10%	0.00%	0.10%	8.48%	15.70%	5.96%	5.55%	11.18%
DS166	paternal	Undetectable	2	166894396	C	T	Exon15	44.46%(11.11%)	52.40%	0.00%(0.00%)	0.10%	2.94%(0.74%)	3.10%	0.01%(0.00%)	0.20%	23.80%(5.29%)	NA	2.27%(0.57%)	NA	NA
DS203	paternal	Undetectable	2	166894440	G	A	Exon15	49.67%	53.52%	0.00%	0.00%	0.00%	0.10%	0.00%	0.10%	0.04%	0.04%	1.27%	0.04%	0.12%
DS312	paternal	Undetectable	2	166900371	GA	—	Exon11	49.87%	43.90%	0.02%	0.30%	13.59%	11.60%	0.02%	0.10%	18.02%	10.48%	11.79%	10.94%	12.31%
DS314	paternal	Undetectable	2	166895938	C	T	Exon14	49.63%	41.40%	0.04%	0.10%	14.36%	7.50%	0.01%	0.00%	25.12%	11.73%	14.02%	18.66%	12.37%
DS296	paternal	Undetectable	2	166904178	C	T	Exon8	49.93%	50.00%	0.00%	0.30%	0.01%	0.00%	0.00%	0.00%	0.31%	NA	NA	NA	NA
DS308	paternal	Undetectable	2	166852541	CT	—	Exon24	49.87%	52.90%	0.00%	0.20%	0.00%	0.30%	0.00%	0.00%	0.03%	NA	NA	NA	NA
DS035	paternal	Undetectable	2	166894440	G	A	Exon15	50.01%	52.24%	0.00%	0.00%	10.24%	15.00%	0.01%	0.20%	NA	NA	NA	NA	NA
DS094	paternal	Undetectable	2	166848852	C	T	Exon26	46.68%(15.94%)	46.10%	0.00%(0.00%)	0.00%	1.33%(0.44%)	1.30%	0.02%(0.00%)	0.00%	NA	NA	NA	NA	NA
DS164	paternal	Undetectable	2	166915194	T	C	Exon2	49.15%	50.00%	0.00%	0.00%	9.32%	8.60%	0.00%	0.00%	NA	NA	NA	NA	NA
DS125	paternal	Undetectable	2	166868765	C	T	Exon19	49.99%	47.23%	0.00%	0.30%	7.15%	6.60%	0.01%	0.60%	NA	NA	NA	NA	NA
DS280	paternal	Undetectable	2	166866246	C	T	Exon20	50.14%	49.50%	0.01%	0.10%	3.52%	4.30%	0.01%	9.70%	NA	NA	NA	NA	NA
DS324	paternal	Detectable	2	166892659	CA	—	Exon16	50.24%	60.35%	0.00%	3.59%	11.01%	15.23%	0.00%	4.15%	NA	NA	NA	NA	NA
DS328	paternal	Detectable	2	166904273	C	T	Exon8	50.16%	49.88%	0.00%	0.01%	29.00%	27.41%	0.01%	0.00%	NA	NA	NA	NA	NA
DS329	paternal	Detectable	2	166911262	C	T	Exon4	50.05%	47.36%	0.00%	0.00%	34.51%	31.37%	0.00%	0.00%	NA	NA	NA	NA	NA
DS004	maternal	Detectable	2	166848782	C	G	Exon26	51.91%(13.02%)	56.10%	0.02%(0.00%)	0.00%	0.00%(0.00%)	0.00%	19.40%(4.87%)	21.20%	NA	17.56%(4.39%)	18.34%(4.58%)	25.60%(6.40%)	17.54%(4.39%)
DS276	maternal	Undetectable	2	166904178	C	T	Exon8	50.07%	49.72%	0.00%	0.30%	0.00%	0.70%	0.82%	1.70%	0.01%	0.73%	0.94%	0.00%	0.10%
DS287	maternal	Detectable	2	166901753	AAGTT	—	Exon10	49.96%	51.70%	0.00%	0.00%	0.00%	0.20%	32.81%	32.90%	NA	35.92%	33.61%	45.65%	39.23%
DS307	maternal	Detectable	2	166905453	A	C	Exon6	49.66%	46.13%	0.00%	0.20%	0.01%	0.00%	25.67%	24.35%	NA	22.16%	NA	8.83%	16.68%
DS128	maternal	Undetectable	2	166868765	C	T	Exon19	49.36%	49.24%	0.00%	0.30%	0.00%	0.10%	13.00%	13.20%	0.01%	10.37%	12.29%	6.24%	0.65%
DS306	maternal	Undetectable	2	166894396	C	T	Exon15	50.00%(12.25%)	48.99%	0.00%(0.00%)	0.10%	0.02%(0.01%)	0.90%	0.06%(0.02%)	0.10%	0.04%(0.01%)	NA	NA	NA	NA
DS136	maternal	Undetectable	2	166859043	G	A	Exon21	49.70%	46.88%	0.00%	0.00%	0.00%	0.30%	11.71%	9.20%	NA	NA	NA	NA	NA
DS152	maternal	Undetectable	2	166898844	C	T	Exon12	49.79%	47.87%	0.00%	0.20%	0.00%	0.10%	2.15%	0.40%	NA	NA	NA	NA	NA
DS316	maternal	Detectable	2	166848864	G	A	Exon26	49.89%	50.70%	0.01%	0.10%	0.01%	0.00%	20.28%	20.30%	NA	NA	NA	NA	NA
DS323	maternal	Detectable	2	166854686	G	A	Exon22	49.79%	47.97%	0.00%	0.10%	0.00%	0.05%	23.14%	23.42%	NA	NA	NA	NA	NA
DS327	maternal	Detectable	2	166894436	C	G	Exon15	49.88%	49.08%	0.00%	0.00%	0.00%	0.00%	24.18%	23.94%	NA	NA	NA	NA	NA

^a^Genomic positions following human reference genome hg19/GRCh37;

^b^Mutant allelic fractions (MAFs) measured by mDDPCR; shown in parentheses are raw data before correction;

^c^ND: sample not detected;

^d^NA: sample not available.

The remaining families (Fig. 1b and Supplementary Fig. S8) showed no mutation signal in either parent’s blood sample–their MAFs could not be distinguished from negative controls, and the lower bound of the 95% confidence intervals of MAFs were lower than the 0.01% cutoff (Fig. 1b). After PASM validation, the MAFs measured by PASM and the corrected MAFs measured by mDDPCR were highly correlated (R^2^ = 0.98, *p* < 2.2e-16 by an F test, Supplementary Fig. S6). The MAFs of mosaic mutations in the parental blood samples ranged from 0.82% to 34.51% (Fig. 1c). Two potential peaks were found at MAFs of 25.0% and 12.5%, suggesting that postzygotic mutations occurred at early stages of embryonic development (Fig. 1e). It is important to note that 15 of the 26 parental mosaicisms (57.69%) detected by mDDPCR could not be detected by conventional PCR and Sanger sequencing (Table 1 and Fig. 1d), demonstrating that using more sensitive technologies in genetic testing and counseling could make it possible to detect cases that would be missed by conventional methods.

Paternity was confirmed for all of the families with parental mosaicism detected by mDDPCR by using STR analysis of six microsatellite markers (Supplementary Table S2 and Supplementary Table S3). Of the 26 parental mosaicisms, 18 (62.1%) were paternal mosaicisms and 11 (37.9%) were maternal mosaicisms. The parent-of-origin sex bias was not statistically significant (*p* = 0.26 by an exact binomial test). One of the 26 mosaic families (DS276, MAF of 0.82% in mother’s blood by mDDPCR) had two non-twin children that inherited the same mutation; allele-specific PCR confirmed that the children’s pathogenic mutations were both inherited from the mosaic mother. One of the 26 mosaic families (DS125) had a pair of monozygotic twins that inherited the same pathogenic mutation from the mosaic father.

### Allele fractions of mosaic *SCN1A* mutations were significantly elevated in paternal sperm

To understand of the relationship between MAFs in sperm and MAFs in blood samples from DS fathers and to directly estimate the potential recurrence risk in the fathers of DS families, purified vital sperm samples from 56 fathers were used for mDDPCR (Supplementary Fig. S7 and Supplementary Fig. S8). A PureSperm 40/80 assay was used to ensure the quality of sperm. mDDPCR was carried out to directly discover mosaic mutations in the paternal sperm samples.

**Figure 2:**
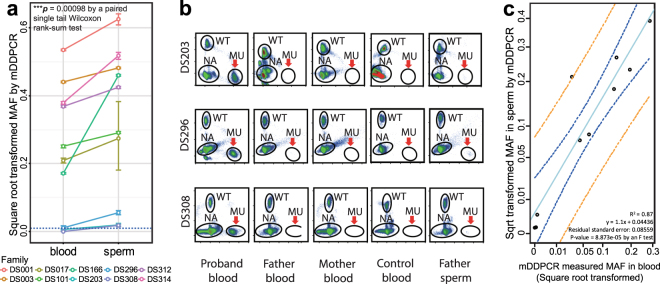
Mutant allelic fractions (MAFs) detected by mDDPCR in paternal sperm samples were significantly higher than those measured in blood. (**a**) MAF in paternal sperm versus blood samples from the same individuals. Each color represents a different father. The MAFs were higher in sperm than in blood, and the difference was statistically significant (*p* = 0.00098 by a paired single-tailed Wilcoxon rank-sum test). In three of the families, only MAFs detected in the sperm sample exceeded the cutoff value of 10^−4^. (**b**) In families DS203, DS296 and DS308, parental mosaicism was only identified in the fathers’ sperm samples and not in the fathers’ blood samples. (**c**) Square-root transformed MAFs measured in parental sperm samples are positively correlated with those in blood (R^2^ = 0.87, *p* = 8.873e-05 by an F test). The 95% CI of the regression line is shown in blue curves; 95% prediction intervals are shown in orange curves.

Ten (17.86% of 56) semen samples were found to carry mosaic mutations corresponding to the proband’s mutation (Table 1 and Fig. 2a). The MAFs ranged from 0.03% to 39.04%. Three paternal mosaicisms with ultra-low MAFs (0.04%, 0.31%, and 0.03%) in families DS203, DS296 and DS308 were detectable in the sperm samples but not in the corresponding blood samples, suggesting that current genetic testing performed in blood samples may have a limited ability to detect mutations (Table 1 and Fig. 2b). These mDDPCR results provided direct evidence for paternal germline-specific mutations leading to Dravet syndrome. For mosaic mutations detected in both sperm and blood, we confirmed that the same mutation was detected (Table 1 and Supplementary Fig. S7). These were postzygotic mutations shared by a proportion of both germline and somatic cells.

Comparison of the MAFs in sperm and in blood samples showed that the sperm samples had consistently higher MAFs than the blood samples, and the difference was statistically significant (*p* = 0.00098 by a paired one-tailed Wilcoxon signed rank test with continuity correction, Fig. 2a). The difference is still significant even after a conditional probability correction that corrects for the fact that the fathers have already transmitted their deleterious mutation to their children, and the children were affected by DS resulting from the heterozygous pathogenic mutations (*p*’ = 0.033, equation (1) and equation (2) in Methods).

The square-root transformed MAF values measured in sperm show a significant positive correlation with the values obtained using blood (*p* = 8.873e-05 by an F test, Fig. 2c) from the same fathers, suggesting that once a mosaic variant has been identified in a DS father’s blood, there is high probability that higher MAFs could be detected in his sperm.

### Mosaic allele fractions were varied across parental peripheral tissues

To investigate the extent to which other parental peripheral tissues may contain the mutant alleles, we collected saliva, buccal epithelium, hair follicles and urine from the parents in 15 families. Thirteen were families with mosaicism detected in parental blood or sperm, and two were families without such mosaicism. mDDPCR showed mutation signals in 97.78% of the peripheral tissue samples (44 of 45) collected from the mosaic parents, and the MAFs were largely similar, although not identical, to the MAFs found in blood (Table 1 and Supplementary Fig. S8). This shows that mosaicism between somatic cells and germline cells is shared in most of the parents. In mosaic parents with MAFs higher than 10^−4^, the mutant alleles could be found in 100% (13 of 13) of the peripheral tissue samples. In 75% of fathers (6 of 8), the MAFs in the sperm were the highest among the available peripheral tissue samples (Table 1). In 40% of mothers (2 of 5), the MAFs in blood were higher than those found in other peripheral tissues (Table 1). No mutations were detected by mDDPCR in other parental tissues from the two families without mosaicism in the blood and sperm (Supplementary Fig. S8).

**Figure 3:**
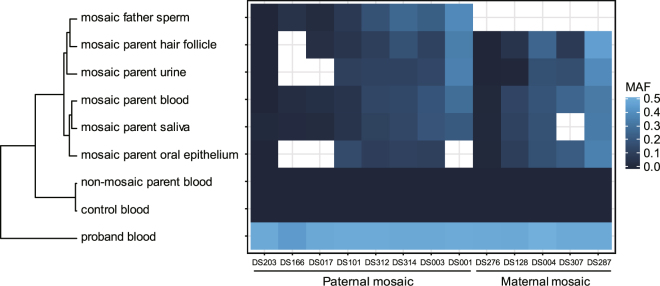
MAFs measured in multiple peripheral tissue samples of the mosaic parents. The color of each cell represents a different MAF. Each column represents a family affected by DS, sorted by the MAFs in the mosaic parents’ blood. Each row represents a tissue or sample type. Hierarchical clustering of square-root transformed MAFs from different sample types shows that samples from the same mosaic parent cluster together, and parental blood and saliva have more similar MAFs than oral epithelia. Hair follicle samples from mosaic parents cluster closer to the paternal sperm samples than to the urine samples. Blood samples from controls and non-mosaic parents all have MAFs of approximately 0% and are clustered together. The heterozygous probands have MAFs of approximately 50%. Multiple tissue and control MAFs analyzed by mDDPCR are shown in Supplementary Fig. S8.

To analyze the relationship between MAFs measured in different tissue samples, we performed hierarchical clustering using the Euclidean distances of square-root-transformed MAFs. Samples obtained from the same mosaic parent clustered together, and they were located on different branches from the probands, non-mosaic parents and clinically normal controls (Fig. 3). Among the parental mosaic samples, peripheral blood and saliva samples showed the greatest similarity of square-root-transformed MAF values, partially because both samples contained considerable proportions of white blood cells. The branch containing blood and saliva was also clustered closely with oral epithelium. Urine samples consisting of urothelia were clustered closer to the branches containing hair follicles and parental sperm than to the branches containing blood, saliva, and oral epithelium. MAFs measured in paternal hair follicles and sperm clustered together, suggesting that hair follicles might be a useful alternative for genetic testing when parental germline cells are unavailable. Interestingly, in one of the three mosaic cases where mutant alleles were found in paternal sperm but not in blood (sperm MAF 0.04%, DS203 father), the mutant allele was also found in three other peripheral tissues, with MAFs ranging from 0.04% to 1.27% (Fig. 3 and Table 1). These results support the idea that a certain fraction of mutant alleles might exist in other tissues, such as the brain, even if they are not detectable in the blood^54^.

### Mosaic parents with epileptic phenotypes had significantly higher mutant allelic fractions than those who are clinically unremarkable

**Figure 4:**
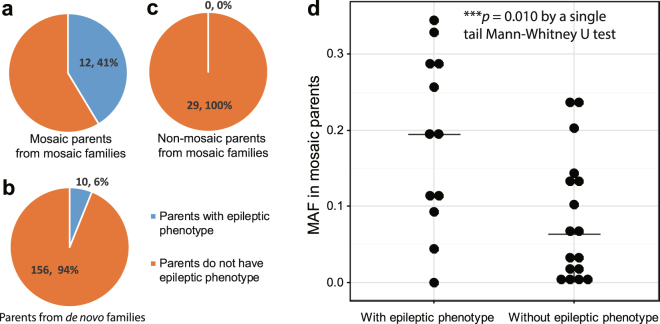
Parental mosaicism contributes to the parents’ epileptic phenotypes. Epileptic phenotype data were collected from all family members by clinicians from Peking University First Hospital. (**a**) Of mosaic parents, 41% report having had an epileptic seizure. Parents with epileptic phenotypes were significantly more likely to be observed among parents with detectable mosaic mutations (odds ratio = 10.8, *p* = 3.0e-06 by a two-tailed Fisher’s exact test). (**b**) Among parents in the “*de novo*” families, 6% have previously had epileptic seizures. (**c**) No non-mosaic parents from mosaic families reported having had any epileptic seizures. (**d**) Among families with detected parental mosaicism, MAFs in the mosaic parents with epileptic phenotypes at any time in their lives were significantly higher than MAFs in epilepsy-free mosaic parents (*p* = 0.010 by a one-tailed Mann-Whitney U test with continuity correction).

To explore the phenotypic contribution of mosaicism in the DS parents, we examined clinical records from the hospital visits and follow-up interviews by telephone and internet for all of the 112 families in the cohort (Supplementary Table S4). Epileptic phenotypes were significantly more likely to be observed in parents with mosaic mutations compared with parents without detectable mutations (odds ratio = 10.8, *p* = 3.0e-06 by a two-tailed Fisher’s exact test, Fig. 4a,b, family scale in Supplementary Fig. S9). Of the mosaic parents from mosaic families, 41% had an epileptic phenotype (Fig. 4a). The percentage of mosaic fathers (44%) with epileptic phenotypes was not significantly different from that of mosaic mothers (36%, odds ratio = 1.4, *p* = 0.72 by a two-tailed Fisher’s exact test, Supplementary Fig. S9).

All non-mosaic parents from mosaic families were symptom-free (Fig. 4c). However, 6% of parents from families regarded as “*de novo*” by mDDPCR were observed to have an epileptic phenotype, indicating that this group falls between mosaic parents and non-mosaic parents and may contain some undetected parental mosaicism (Fig. 4b). Among the 29 families with detected parental mosaicism, mosaic parents with an epileptic phenotype had significantly higher MAFs than those without (*p* = 0.010 by a single-tailed Mann-Whitney U test with continuity correction, Fig. 4d). Distribution of the variants on the SCN1A protein showed that mosaic variants were less frequently observed in transmembrane alpha-helix regions but more frequently observed in intra- or extracellular coil regions (Supplementary Fig. S10). Variants from mosaic parents with an epileptic phenotype had more significant effects on coil formation than those from mosaic parents without an epileptic phenotype (*p* = 0.022 by a single-tailed Mann-Whitney U test, Supplementary Fig. S10).

### Detectable mosaicism directly causes DS and influences the phenotype

**Figure 5:**
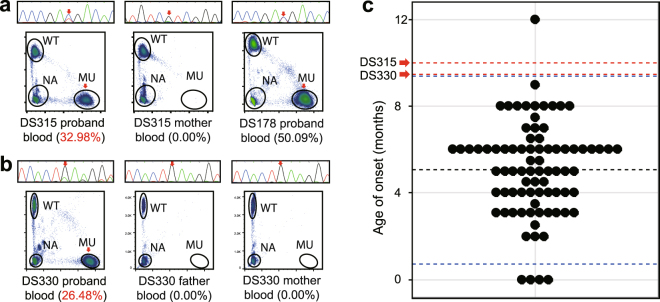
Proband mosaicism is confirmed and influences phenotypic characters. (**a**) The proband from the DS315 family turned out to be a mosaic proband with an MAF of 32.98% after homology correction. Proband DS178 and DS315 share the same point mutation, NM_001165963.1: c.1837C > T. DS178 had an onset of seizures at 6 months of age; however, the mosaic proband DS315 has his first seizure at 10 months. (**b**) The proband from the DS330 family was found to have an MAF of 26.48% after homology correction. No mutation signals were found in his parents. (**c**) The distribution of the ages of onset for all other probands carrying *de novo SCN1A* mutations are shown; arrows on dashed red lines indicate that the ages of onset of DS330 (9.5 months, *p* = 0.04816 by a single-tailed Wilcoxon rank sum test with continuity correction) and DS315 (10 months, *p* = 0.04816 by a single-tailed Wilcoxon rank sum test with continuity correction) are significantly later than those of nearly all probands carrying other *de novo SCN1A* mutations. Dashed blue lines describe the 95% confidence intervals of the distribution of proband ages of onset.

In our cohort, two DS probands were identified as carrying *SCN1A* mutations as mosaics, including DS315 with an MAF of 32.98% (Fig. 5a) and DS330 with an MAF of 26.48% (Fig. 5b). Compared to other probands with heterozygous mutations, these two mosaic probands had their first seizure onset at the ages of 9.5 and 10.0 months, which were significantly later than the ages of onset of probands with other *SCN1A* variants (N = 80, *p* = 0.04816 and 0.04816 by a single-tailed Wilcoxon rank-sum test with continuity correction, Fig. 5c). In particular, the age of onset of proband DS178, who was heterozygous for the same substitution as the mosaic proband DS315 (NM_001165963.1: c.1837C > T), was six months, not significantly different from that of the heterozygous probands with other mutations (N = 81, *p* = 0.64 by a two-tailed Wilcoxon rank-sum test with continuity correction). The results demonstrated that the differences in age of onset were not related to variant differences. These results again suggest that differences in MAFs may contribute to phenotypic severity.

## Discussion

In this study, we detected mosaicism and quantified the MAFs of mutations in blood and tissue samples, including paternal sperm. This study was conducted in a large Chinese DS cohort (Fig. 1), and a relatively high proportion of parental mosaicism was identified (25%, 26/112). Compared with the postzygotic single-nucleotide mosaicisms identified and validated in parents from families with polygenic or complex disorders such as autism spectrum disorders^14–17^, intellectual disability^18^, and epileptic encephalopathies^19^, we also found in our DS cohort that a relatively high proportion of parents are carrying mosaic mutations even though their children’s cases were previously regarded as “*de novo*” (Fig. 1). According to the benchmarking test (Supplementary Fig. S4), Sanger sequencing could only detect candidate mutations with MAFs greater than 5%, which matched a previous report^7^. PASM could detect MAFs as low as 0.5%, which matched our previous benchmark results^21^. The mDDPCR detection limit in our study was 10^−4^, similar to the previously reported limit in cancer samples^50^. mDDPCR has the most accurate mosaic detection limit and is performed with single-molecule resolution. In this study, over 55% of cases of parents carrying mosaicism were detected by mDDPCR and could not be detected by conventional PCR with Sanger sequencing (Table 1). This demonstrates the importance of using more sensitive technologies in clinical genetic testing. Further experimental validation using multiple ultra-sensitive NGS approaches, such as o2n-seq^55^ or duplex sequencing^56^, might help to confirm the MAFs measured by mDDPCR at different levels of sensitivity.

Compared with other monogenic childhood neurological diseases with reported cases exhibiting deleterious mosaic mutations, such as Rett syndrome (caused by mutations in *MECP2)* ^57–59^, epilepsy in females with mental retardation (caused by mutations in *PCDH19)* ^39,40,60^, mosaicism related to *ATP1A3* ^37^ or epilepsy-related neurodevelopmental disorders^13^, our DS cases with *SCN1A* mosaicism exhibit milder phenotypes compared with all the other probands who were detected with heterozygous mutations in *SCN1A* (Fig. 5). However, their phenotypes still met all of the diagnostic criteria for DS. The mosaic mutations in these disorders also seem to be more frequently observed sporadically rather than being clustered in mutation hotspots, which is in accordance with the fact that *SCN1A* mutations do not cluster in mutation hotspots.

We found elevated *SCN1A* pathogenic mutant allele fractions in the mosaic fathers’ sperm compared to their blood samples (Fig. 2a). In three families, the mutant alleles were undetectable only in the fathers’ peripheral blood samples. These findings are in agreement with previous publications that reported changes in the mutation spectrum and mutation rate in parental germline cells^22,38^, and they demonstrate the importance of including paternal sperm samples in genetic testing. Germline mutations detected in the fathers of probands affected by diseases caused by cancer-related genes, such as Apert syndrome (caused by *FGFR2* mutations), Costello syndrome (caused by *HRAS* mutations), and aggressive thyroid cancer syndrome (caused by *MEN2B* mutations)^25–27,61^, have been previously studied. As in those studies, they found an accumulation of mosaic mutations and an elevation of the MAF in germline cells. A spermatogonial selection theory has been previously proposed to explain these observations^25–27^. To our knowledge, our study is the first to demonstrate this phenomenon for any neurological disorder at a cohort level, and our results indicate potential spermatogonial selection in epileptic neurological disorders such as DS. Interestingly, a previous study of healthy human tissue also found *SCN1A* mutations in their list from the Supplemental Information^62^, although the gene was not incorporated into their report.

Purified vital sperm samples reflect the potential sperm population that could contribute to offspring. Our results provide direct evidence that mutations in the paternal germline can contribute to the elevated recurrence risks observed in families with detectable mosaic mutations (Fig. 2). The mother DS276M was found to have a mosaic mutation and transmitted the same mutant allele to two non-twin children. Mothers from mosaic families DS001, DS128, and DS296 were pregnant with their second child. Prenatal testing found that the fetus from family DS128 had a heterozygous deleterious mutation in *SCN1A* (NM_001165963.1: c.3733 C > T), which was the same as the proband and the mosaic mother of the family. The other two fetuses from DS001 and DS296 were free of pathogenic mutations. These results demonstrate that there is a significantly increased disease recurrence risk for these mosaic families. It is also important to note that although we found higher MAFs in paternal germ cells than in paternal blood, paternal mosaicism is not invariably observed in fathers, and we think this is an important difference between parental mosaicism and “*de novo*” mutations affecting single germ cells^49,63^ in common neurological disorders: compared with “*de novo*” mutations parental mosaicism would significantly increase the recurrence in the mosaic family and the two different groups of mutations might undergo different selective pressures.

The overall high correlation of MAF values between parental tissues (Fig. 3) suggests that the mutations detected in this study occurred in early stages of development^64^, whereas germline-elevated mosaicism contributes to the elevated genetic transmission risks in DS families. Postzygotic mosaicisms led by various types of mutations have been systematically studied in samples collected from fetal^64^ or postnatal tissues^34,41,65,66^, and data from those studies also support the idea that certain somatic mutations occur in early stages and are present in multiple tissues, whereas mutations occurring at later stages could affect cells in the brain but be undetectable in other tissues, especially in neurological disorders^3,4,49^. In our cohort, parents with epileptic phenotypes were significantly more invariably observed among those with mosaic genotypes (Fig. 4a,c). This indicates that there is mosaicism in the central nervous systems of these parents. The MAFs measured in paternal sperm are equal to the proportion of sperm cells carrying the *SCN1A* mutant allele, and exactly the same allele in the proband causes the disease; therefore, our measurement of the MAFs in sperm provide an estimate of the probability that the father will transmit a deleterious allele to another child. Thus, measurements of the frequencies in sperm provide important information for clinicians.

Mosaic parents with epileptic phenotypes have significantly higher MAFs, and their mutations influence coil formation (Fig. 4d), which confirms our previous findings^21^. Functional predictions for mutations in probands from the mosaic families and “*de novo”* families show similar deleterious probabilities (Supplementary Fig. S10). Approximately 60% (56% of fathers and 64% of mothers, Supplementary Fig. S9) of mosaic families did not have any parents with epileptic phenotypes, which again demonstrates the importance of detecting mosaicism in symptom-free parents using ultra-sensitive technology. Altogether, our results and follow-ups have shown that genetic testing with enhanced detection sensitivity can provide parents with more informative genetic counseling recommendations^21,55,64,66^. We suggest the use of more sensitive technologies, the use of paternal sperm samples, and the use of multiple parental peripheral tissues in clinical genetic testing for monogenic disorders. We also recommend studying mosaicism in the germline samples from other rare and common disorders.

## Subjects and Methods

### Description of the DS cohort and diagnostic criteria

A total of 719 Chinese DS probands from 2005 and later were collected from the child neurology units of Peking University First Hospital. Sanger sequencing, panel NGS sequencing and MLPA identified 591 (82% of 719) of the probands as carrying potentially pathogenic *SCN1A* mutations (rare missense, nonsense, frame-shift, and splice site mutations)^20,21,59,44^. All probands fulfilled the following criteria and were diagnosed with DS^21,43,44,48,67^: (a) seizure onset within 12 months of birth (average age of onset of 5.17 months, 95% CI 5.17 $\mp$ 4.42 months), with the first event often being a fever-induced seizure (FS); (b) normal early development; (c) prolonged generalized or hemiclonic seizures that were often triggered by fever; (d) in additional to FS in the first year of age, multiple seizure types (myoclonic, focal, atypical absences) occurring after 12 months; (e) psychomotor developmental delay after 12 months with possible ataxia and pyramidal signs; (f) normal interictal electroencephalography in the first year of life followed by generalized, focal, or multifocal discharges; and (g) pharmaco-resistant seizures.

Phenotypic diagnoses and clinical follow-ups were carried out by clinicians from the Department of Pediatrics, Peking University First Hospital. All studies were approved by the Institutional Review Board at Peking University (IRBPU) and the Ethics Committee of Peking University First Hospital under the approval number IRB00001052-11087. Written informed consent was provided by participants or their statutory guardians before enrollment. All methods from this study were performed in accordance with the relevant guidelines and regulations of the IRBPU.

### DNA isolation, *SCN1A* mutation screen and mosaicism screen

Blood DNA was extracted in the Central Laboratory of Peking University First Hospital. A PureSperm 40/80 assay (Nidacon) was used for the purification of vital sperm from paternal semen samples; DNA was extracted from purified sperm using a phenol-chloroform extraction method. To avoid contamination of low-fraction genomic mutant alleles from the proband, DNA samples from paternal sperm and parental tissues were extracted separately in the Human Genetic Resources Core Facility of Peking University. Different tissue samples, including paternal semen, parental saliva, buccal epithelium, hair follicles and urine, were collected, and DNA was extracted according to recommended protocols of the QIAamp DNA micro kit (Qiagen) or TIANamp micro DNA kit (Tiangen). *SCN1A* mutations were first screened in all blood samples by Sanger sequencing or captured in epilepsy panel sequencing (MyGenostics). mDDPCR analysis was carried out to measure the mutant allele fractions in all available samples in probands and their parents. Seventy-nine parental blood samples were examined with the amplicon resequencing method PASM^21^. Detailed DNA isolation, *SCN1A* mutation screening and mosaicism screening protocols are provided in the Supplementary Methods.

### Framework for mDDPCR analysis

Single-molecule mDDPCR analysis was introduced for the absolute quantification of MAFs in the *SCN1A* mutated DS cohort. The details for the mDDPCR analysis are provided in Supplementary Fig. S2 and the Supplementary Methods. TaqMan MGB probes labeling the mutant allele with the FAM fluorophore and the wild type allele with the VIC fluorophore (P/N:4331349, Applied Biosystems by ThermoFisher) were designed and ordered from ThermoFisher. Genotyping reactions to test the TaqMan assay specificity were carried out on a StepOnePlus real-time system (Applied Biosystems by ThermoFisher, Supplementary Fig. S3). Genomic DNA was sheared to a peak length of 3000 base pairs using an M220 ultrasonicator (Covaris). To minimize the potential contamination of low-fraction mutant alleles, DNA from multiple tissues was sheared separately. The ultrasonicator was treated with ultraviolet radiation or DNAZap after shearing the DNA of each proband. Emulsions were generated by a Raindrop Source emulsion generator (RainDance). To balance the amplification efficiency, a ramp-temperature-controlled (0.6 °C/s) PCR amplification was carried out on an ETC-811 thermocycler (EASTWIN). Droplet detection was carried out on a Raindrop Sense emulsion detector (RainDance).

### Validation of parental mosaicism candidates using PASM

To confirm the mosaicism detected by mDDPCR, blood DNA samples from mosaic candidates were also examined using an amplicon-based deep-resequencing method that we had previously published, PASM^15,21^. A region of approximately 400 base pairs around the candidate mutation sites was amplified from the blood samples of the probands, their parents and the negative controls. Amplicons were independently barcoded before semiconductor sequencing using a 318 chip on a PGM sequencer or a 530 chip on an S5 sequencer (ThermoFisher). Pileup bam files aligned to hg19 were processed using a hierarchical Bayesian model described in our previous publications^20,21,68^ to estimate MAFs with the maximum posteriori and the 95% credible intervals (CI) for PASM-estimated MAFs. Primers for PASM detection are provided in Supplementary Table S5.

### Sequential dilution benchmarking for the detection limit of mDDPCR, PASM and Sanger sequencing

We used a sequential dilution-based benchmarking test and compared the detection limits of mDDPCR, PASM and conventional PCR with Sanger sequencing for the quantification of MAFs. Blood DNA from the proband of family DS308 (NM_001165963.1:c.4562_4563del) was regarded as a 50% MAF standard and was sequentially diluted to provide DNA standards with theoretical MAFs of 5%, 0.5%, 0.05%, 0.005%, 0.0005% and 0.00005%. The samples were diluted with negative control blood (ACC1)^20,68^. The sequential dilution standards were measured by using different mutation detection approaches such as mDDPCR, PASM and Sanger sequencing. Two replicates were carried out for control samples and for standards with theoretical MAFs lower than 0.05%.

In the sequential dilution benchmark, Sanger sequencing could only detect candidates with MAFs greater than 5%. PASM could detect MAFs as low as 0.5%. mDDPCR could detect MAFs as low as 0.005%, and the flow cytometry scatter plot of mDDPCR showed gradually decreasing amounts of mutant droplets (Supplementary Fig. S4).

To determine the threshold for positive mosaic cases, we evaluated the performance of different TaqMan assays for mDDPCR using negative control DNA samples. Based on these results, we determined that a mosaic mutation was considered detected if the 95% CI lower bound of the binomial parameter estimation of its MAF was greater than or equal to 10^−4^ (Supplementary Fig. S4 and Supplementary Methods).

### Correction based on genomic similarity

The genomic sequences of *SCN1A* exon9, exon15, and exon26 are located within regions that are highly similar to other genomic regions (Supplementary Table S6). Thus, the ~130 bp TaqMan targeted sequences might detect false positive in the probands or their parents. To resolve this problem, we used BLAST and BLAT to identify similar genomic regions (Supplementary Fig. S5), and we corrected mDDPCR results according to the number of similar sequences detected by BLAST and BLAT (Supplementary Table S6). The corrected mDDPCR MAFs were in strong accordance with the PASM results (R^2^ = 0.98, p-value < 2.2e-16 by an F test, Supplementary Fig. S6).

### Paternity test for mosaic positive families

Paternity testing was carried out for all mosaic candidate families. Six informative microsatellite markers, AFMa081we1, D2S2157, D2S124, D2S2363, D2S1395, and D2S1379 (STR information provided in Supplementary Table S2), were selected for linkage analysis at the *SCN1A* locus (2q24.3). Previously published protocols were followed^45^. Genotypes were analyzed with GeneMarker V2.2.0 (SoftGenetics).

### Functional predictions for *SCN1A* variants

The functional effects of all validated nonsynonymous variants were predicted by using integrated functional inference of SNVs in human (iFish)^46^. For frameshift indels, the deleterious probabilities were set to 100%. Population single nucleotide polymorphism (SNP) data for *SCN1A* genomic sequences were downloaded from the Exome Aggregation Consortium (ExAC), and 1% was used as the cutoff for common SNPs in the *SCN1A* genomic region^61^.

### Conditional probability correction for mutation transmission and the higher MAFs in sperm than in blood

For all of the fathers, the probability of observing a higher MAF in sperm than in blood under the condition that their mutations had already been transmitted to their children at the probability of the MAF in their sperm was considered by using the equation (1) to calculate conditional probability:

1 $$\begin{array}{ccc}{P}_{sp|T} & = & \frac{{P}_{sp,T}}{{P}_{T}}\\  & = & \frac{{P}_{T|sp}\,\cdot \,{P}_{sp}}{{P}_{T|sp}\cdot {P}_{sp}\,+\,{P}_{T|bl}\,\cdot \,{P}_{bl}}\\  & = & \frac{MA{F}_{large}\,\cdot \,0.5}{MA{F}_{large}\,\cdot \,0.5\,+\,MA{F}_{small}\,\cdot \,0.5}\\  & = & \frac{MA{F}_{large}}{MA{F}_{large}\,+\,MA{F}_{small}}\end{array}$$

Here, *P*_*T*_ is the probability of transmitting a deleterious mutation to a child, *P*_*sp*_ is the probability of observing an MAF in sperm higher than the MAF in blood, and *P*_*bl*_ is the probability of observing an MAF in sperm lower than the MAF in blood. *P*_*sp|T*_ is the probability of observing an MAF in sperm higher than the MAF in blood given that the mutation has already been transmitted to the offspring. *P*_*sp,T*_ is the probability of observing an MAF in sperm lower than the MAF in blood and observing that the mutation has already been transmitted to the offspring. *P*_*T|sp*_ is the probability of observing transmission of the mutant allele to the offspring given that the MAF in sperm was measured to be higher than the MAF in blood, *P*_*T|bl*_ is the probability of observing transmission of the mutant allele to the offspring given that the MAF in sperm was measured to be higher than the MAF in blood. *MAF*_*large*_ is the larger of the MAFs measured in paternal blood and sperm, and *MAF*_*small*_ is the smaller of the MAFs measured in paternal blood and sperm.

For all blood-sperm sample pairs from the mosaic fathers, the corrected conditional probability *P*_*corrected*_ of observing *MAF*_*sperm*_ < *MAF*_*blood*_ given the transmission to children could be calculated. The probability that in all samples, sperm MAFs were observed to be higher than blood MAFs from the ten observations given that they all showed transmission of the mutant allele to the affected children was calculated using equation (2). This corresponds to the single-tailed probability of a test of *MAF*_*sperm*_ > *MAF*_*blood*_ given the condition that each parent transmitted the mutant allele to their affected child.

2 $$\begin{array}{ccc}{P}_{corrected} & = & \prod _{i=1}^{n}{P}_{i,sp|T}\\  & = & \prod _{i=1}^{n}\,\frac{MA{F}_{i,large}}{MA{F}_{i,large}\,+\,MA{F}_{i,small}}\end{array}$$

After calculating the real data for the $i$th father, the corrected *p′* equals 0.033, which means that *MAF*_*sperm*_ > *MAF*_*blood*_ in mosaic fathers is significant after correction for transmission.

### Data availability

Raw mDDPCR flow cytometry signal files are provided at https://pan.baidu.com/s/1mi5O4HE with access code nk7s. Sequencing data are available on SRA under the accession number SRP105250.

### Code availability

Software versions and web resources are provided in Supplementary Table S7 and the Supplementary Information, R package for the Bayesian model of PASM is available at https://github.com/Yyx2626/yyxMosaicHunter.

## Electronic supplementary material

Supplementary information
