# Supplementary material for: Genomic mosaicism in paternal sperm and multiple parental tissues in a Dravet syndrome cohort
Source: Sci Rep. 2017 Nov 15;7:15677. doi: 10.1038/s41598-017-15814-7 (PMC5688122; doi:10.1038/s41598-017-15814-7)
Supplement: Supplementary file 1 — Supplementary information [file 41598_2017_15814_MOESM1_ESM.pdf]

Genomic mosaicism in paternal sperm and multiple parental tissues in a Dravet syndrome cohort

Xiaoxu Yang[1,+], Aijie Liu[2,+], Xiaojing Xu[2], Xiaoling Yang[2], Qi Zeng[2], Adam Yongxin Ye[1,3,4], Zhe Yu[3,5], Sheng Wang[6,7], August Yue Huang[1], Xiru Wu[2], Qixi Wu[3,5,*], Liping Wei[1,*], & Yuehua Zhang[2,*]

1 Center for Bioinformatics, State Key Laboratory of Protein and Plant Gene Research, School of Life Sciences, Peking University, Beijing 100871, China

2 Department of Pediatrics, Peking University First Hospital, Beijing 100034, China

3 Peking-Tsinghua Center for Life Sciences, Beijing 100871, China

4 Academy for Advanced Interdisciplinary Studies, Peking University, Beijing 100871, China

5 School of Life Sciences, Peking University, Beijing 100871, China

6 National Institute of Biological Sciences, Beijing 102206, China

7 College of Biological Sciences, China Agricultural University, Beijing 100094, China

+ These authors contributed equally to this work.

* Present address: Department of Pediatrics, Peking University First Hospital, 8th Xi'anmen Avenue, Beijing 100034, China (Y.Z.); Room 307, Center for Bioinformatics, Wangkezhen Building, 5th Yiheyuan Road, Peking University, Beijing 100871, China (L.W.); Room 342, School of Life Sciences, Wangkezhen Building, 5th Yiheyuan Road, Peking University, Beijing 100871, China (Q.W.).

Correspondence and requests for materials should be addressed to Emails: zhangyhdr@126.com (Y.Z.); weilp@pku.edu.cn (L.W.); wuqx@pku.edu.cn (Q.W.)

Supplementary Information

Supplementary methods

Parental tissue DNA sample isolation and genetic analysis. Lymphocyte genomic DNA of the peripheral blood samples from the families were extracted using a salting-out procedure[1] or the TIANamp genomic DNA kit (Cat No: DP304, Tiangen Biotech, Beijing, China) following the manufacturer's recommended protocol.

Genomic DNA samples from one swab of buccal epithelium, 10-15 hair follicles and 20-30 mL of urine from the parents were isolated with the QIAamp DNA micro kit (Cat No: 56304, Qiagen, Hilden, Germany) or TIANamp micro DNA kit (Cat No: DP316, Tiangen Biotech, Beijing, China) following the manufacturer's instructions.

Genomic DNA samples from 2 mL of saliva were collected and extracted with the Aidlab saliva genomic DNA kit (Cat No: DN0803, Aidlab Biotechnologies, Beijing, China) or TIANamp micro DNA kit (Cat No: DP316, Tiangen Biotech, Beijing, China) following the manufacturer's recommended protocols.

To eliminate the influences of lymphocytes and contamination from seminal plasma, vital sperm was purified from fresh semen (within 1 hr. of collection) using a PureSperm gradient separation kit (PureSperm 40/80, Nidacon International, Gothenburg, Sweden). Genomic DNA from purified sperm was extracted by using a phenol-chloroform extraction method and ethanol precipitation. Genomic DNA from a frozen whole semen sample was extracted with the TIANamp genomic DNA kit (Cat No: DP304, Tiangen Biotech, Beijing, China).

SCN1A mutations were first screened in all families using peripheral blood samples by Sanger sequencing or captured epilepsy panel NGS sequencing. End-point genotyping qPCR and Raindrop Micro-droplet digital PCR (mDDPCR) analysis were carried out for all samples from all families, and the same mutation was screened in probands, their parents and the negative controls. Seventy-nine of 112 families' parental blood samples were examined with the amplicon resequencing method PASM.

Framework for mDDPCR analysis. Single-molecule mDDPCR analysis was used for the absolute quantification of MAFs in the *SCN1A* mutation DS cohort. The framework of mDDPCR analysis is described in Supplementary Fig. S2. Customized TaqMan genotyping assays (Part No: 4331349, Applied Biosystems by ThermoFisher, Foster City, CA, USA, assay IDs are provided in Supp. Fig. S3) including duplex MGB probes labeling mutant alleles with FAM fluorophores and wild type alleles with VIC fluorophores were designed based on the Sanger screening results.

An end-point genotyping experiment was first carried out to test the specificity and performance of each TaqMan assay (Supplementary Fig. S2) on an ABI Real-time PCR system (StepOne Plus, Applied Biosystems by ThermoFisher, Foster City, CA, USA). Samples of a proband, two parents, a normal control and NTCs were prepared, and 2-3 replicates were carried out for each genotyping reaction. Each 20 uL qPCR reaction contains 10 uL of 2X TaqMan genotyping master mix (Part No: 4381656, Applied Biosystems by ThermoFisher, Foster City, CA, USA), 1 uL TaqMan genotyping assays and 10 ng peripheral blood genomic DNA as well as 6 uL nuclease-free water (Part No:

4387936, Ambion by ThermoFisher, Austin, TX, USA). Assays that generated signals for both alleles in the proband samples but generated mostly signals for wild type alleles in the controls and parents were selected for the remaining mDDPCR reactions. Assay IDs and assay indexes are listed in Supplementary Fig. S2.

DNA samples were first sheared to a peak length of 3 kb using a focused ultrasonicator (M220, Covaris Inc., Woburn, MA, USA) to avoid disruption of the pico-droplet emulsion by following the manufacturer's recommended protocol. For tissue samples with low DNA concentrations (urine and hair follicles from certain donors that yielded less than 10 ng/uL in the extracted samples) or samples dissolved in high concentrations of TE/AE buffer, another purification process was carried out using 2X volumes of AMPure XP beads (Part No: A63882, Agencourt by Beckman Coulter, Brea, CA, USA).

qPCR reactions were then transferred to new tubes for mDDPCR analysis, and the PCR reactions were carried out in 60 uL. These procedures guaranteed that the number of occupied droplets would be under 5% of the total generated droplets. A total of 2.4 uL 25X droplet stabilizer (Ref No: 30-07026, RainDance Technologies, Boston, MA, USA), 3 uL TaqMan genotyping assay, 30 uL genotyping master mix and approximately 500 ng genomic DNA (dissolved in nuclease-free water after buffer exchange) were added to each reaction system for emulsion generation in each channel of a Raindrop Source emulsion generator (Raindrop Source, RainDance Technologies, Boston, MA, USA).

Amplification reactions were carried out with an ETC-811 thermocycler (EASTWIN Inc., Beijing, China). A 98 ℃ incubation was carried out for 20 min after the recommended TaqMan genotyping PCR procedure to denature the polymerase. The RAMP rate of the reactions was set at 0.6℃/s to ensure sufficient amplifications for the millions of droplets. After amplification, the emulsions were transferred directly to the droplet detector (Raindrop Sense, RainDance Technologies, Boston, MA, USA), and signals from the different photomultiplier tubes (PMTs) were detected and recorded.

Raw fcs files were analyzed using Raindrop Analyst v3 software. The relative positions of signals of the mutant allele and the wild type allele were first determined by using the positive and negative controls. The compensation process was carried out by using the wild type (WT), mutant (MU) and not amplified (NA) signal clusters from the positive controls. The compensation matrix was used to rotate the signals on the axes. Relative positions were then applied to the other samples. Empirical adjustments was carried out on these samples to ensure the correct location of different signal clusters. From the number of signals from wild type and mutant droplets, 95% confidence intervals for the mutant allele fractions were calculated based on a binomial distribution.

Serial dilution benchmarking for the detection limit of mDDPCR. The detection limit for mutation allelic fraction (MAF) is approximately 10^{-1} for a high-definition melting curve using mismatch amplification mutation assays[2] and pyrosequencing[3], and the limit is 10^{-2} for TaqMAMA[4] and denaturing HPLC[5]. Recently developed next-generation sequencing (NGS) approaches, including molecular inversion probes (MIPs)[6], rolling circle amplification (RCA)[7], duplex sequencing[8] and maximum-depth sequencing (MDS)[9], can identify mutations

with MAFs much lower than 10^{-3}. The next-generation digital PCR technology, (mDDPCR), offers an ultra-sensitive and cost-effective alternative; it can generate up to 10 million droplets in an emulsion system[10-12] and can theoretically detect mutations with MAFs of 10^{-4} or lower[13-16]. To confirm the detection limits of mDDPCR, PASM and PCR with Sanger quantification for MAF measurements in blood DNA samples, a serial dilution benchmarking test was carried out. Blood DNA from a positive blood sample of the DS308 family (NM_001165963.1: c.4562-4563del), regarded as a 50% MAF carrier, was sequentially diluted using the same serial dilution standards. The positive sample was serially diluted by using a negative control blood DNA sample with the same DNA concentration estimated by Qubit. The sequential dilutions generated artificial standards with MAFs of 50%, 5%, 0.5%, 0.05%, 0.005%, 0.0005% and 0.00005%. The serial diluted standards had the same concentration of 20 ng/uL. A total of 500 ng DNA was used from each standard, and the negative control was used for mDDPCR examination. A total of 20 ng DNA from each sample was used as a template for PCR with Sanger sequencing and for PASM. Detection of the same standards using different MAF detection approaches could help to better determine the detection limits of these methods.

The log-transformed theoretical MAFs of the standards, the MAFs, and the 95% binomial confidence intervals detected by Raindrop mDDPCR are plotted in Fig. 1a. For theoretical samples with MAFs higher than 0.005%, a high linear correlation was found between the known standards and the mDDPCR results ($R^2 = 0.98$ for MAF 50% to 0.005% on the log scale). Sanger chromatograms, PASM MAFs and 95% CIs are presented near each data point. Using the serial dilution benchmarks, we were able to identify candidate cases of mosaicism by Sanger sequencing with MAFs over 5%, which matches previous reports[4]. PASM was able to detect MAFs as low as 0.5%, which matches our previous benchmark[17]. Raindrop mDDPCR could detect an MAF of 0.005%, and a flow cytometry scatter plot of the mDDPCR results showed a gradient of densities in the mutant droplets (Supplementary Fig. S3); the detection limit was similar to a previously reported limit in cancer samples[13], and it was more sensitive than NGS approaches.

After evaluating the performance of different TaqMan assays for mDDPCR with negative control blood samples, mDDPCR was simulated under different sample inputs (Supplementary Fig. S3). The upper bounds of 75% binomial CIs for the WT and MU droplet numbers in negative control reactions were first estimated (Supplementary Fig. S3, black points); the empirical error rate was then calculated as $2.2*10^{-5}$. Under this null model, the upper bound of the 95% binomial CI under different total templated droplets (WT+MU) was calculated (Supplementary Fig. S3, gray points). An MAF of 10^{-4} was set as the cutoff for the 95% CI lower bound of parameter estimation (Supplementary Fig. S3, blue horizontal line) for mDDPCR results when the number of templated droplets was greater than 10^4. This cutoff for mosaicism is stricter for larger amounts of sample input.

Correction based on genomic similarity. In some parts of the *SCN1A* genomic region, there are sequences that are highly similar to other genomic regions. For the ~130 bp TaqMan targeted sequences, this might lead to false positive detection of mosaicism in both the proband and parent. The PASM assay used 400 bp amplicons or ~1000 bp nested amplicons. Higher fractions of mutant alleles in the Sanger sequencing results for

heterozygous probands made it possible to overcome this problem[17].

In the mDDPCR analysis, parental and child mosaicism rarely occurred in the same family, so we used BLAST and BLAT to identify genomic regions that were similar to some of the exons of *SCN1A*. Supplementary Table 1 shows uncorrected mDDPCR and PASM results for 37 DS probands. The results show that parts of exon 9, exon 15 and exon 26 of *SCN1A* are highly similar to *SCN2A*, *SCN3A* and *SCN9A* (Supplementary Fig. S4). In certain regions, the sequence similarity reached 100% according to BLAST and BLAT in GRCh38 and hg19.

For the short amplicons designed for the TaqMan qPCR assays, non-unique regions might be hard to avoid. In one of these non-unique regions, a heterozygous proband can be found to have a mutant allele fraction of approximately 1/4 (Supplementary Fig. S4). In two regions, the MAF is 1/6 (Supplementary Fig. S4), and in three regions, it is 1/8 (Supplementary Fig. S4). Using longer amplicons that could extend beyond exon boundaries might reduce the bias.

After identifying highly similar regions for each variant, we corrected the other mDDPCR results according to the heterozygous proband's mutant allele fraction detected by the same set of assays. Correlations between uncorrected mDDPCR and PASM were relatively weak (R^2 = 0.83, Supplementary Fig. S5). The corrected values were in strong accordance with the PASM results (R^2 = 0.98, Supplementary Fig. S5). Correction based on genomic similarity guaranteed high correlations between mDDPCR and PASM.

The versions of the software used in this study are listed in Supplementary Table S6.

Supplementary Figures

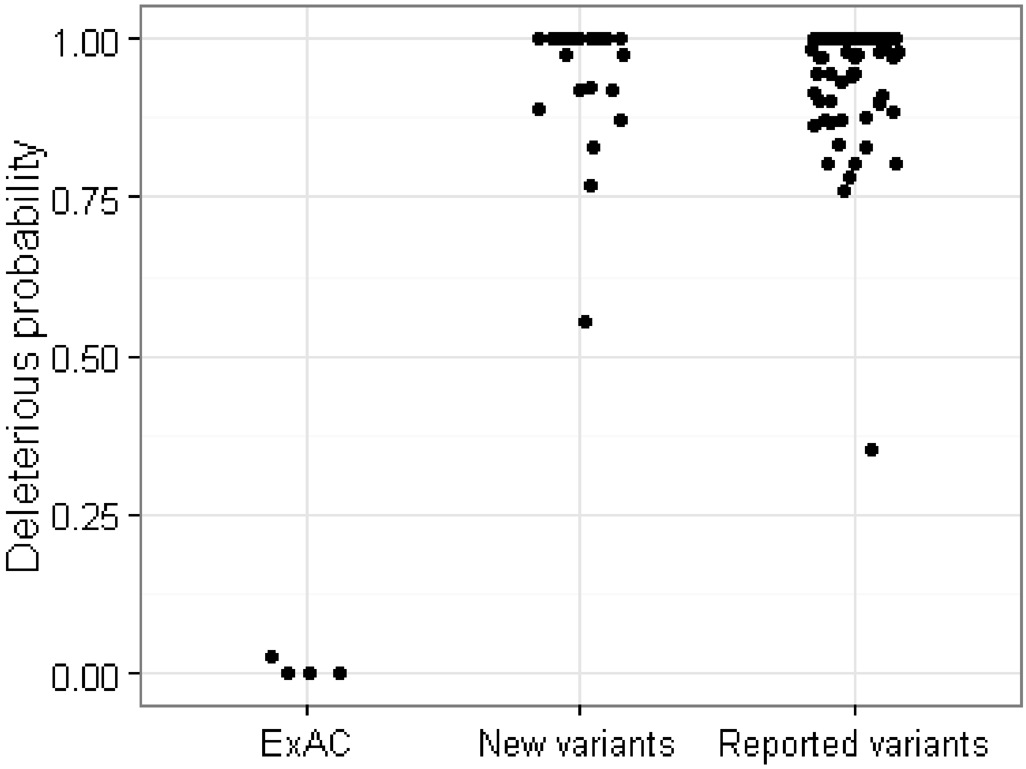

Figure S1. Functional predictions for new and reported deleterious variants detected in the DS probands.

According to the predictions of deleterious effects by iFISH, the new variants identified in this study have similar predicted deleterious effect probabilities as variants that have already been reported. All potential disease-causing variants show significant differences from neutral missense variants recorded in ExAC.

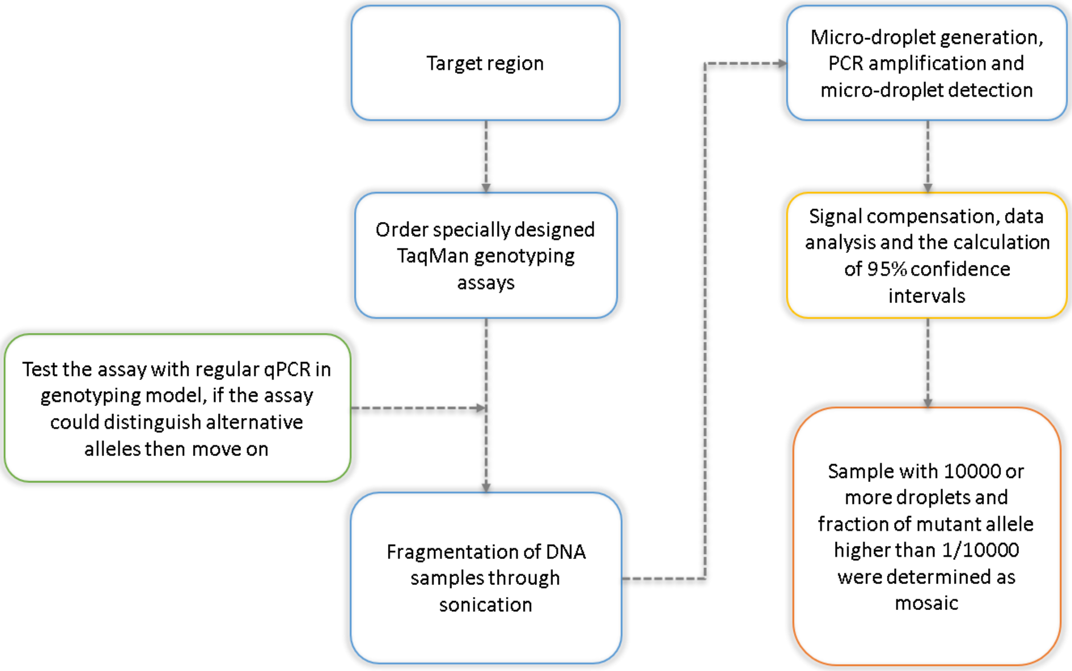

Figure S2. Pipeline for mDDPCR analysis for *SCN1A* mutated DS families.
Target regions were identified based on the probands' direct Sanger sequencing data. Target regions based on the Sanger screening results were obtained from the UCSC genome browser (http://genome.ucsc.edu) based on hg19 coordinates. TaqMan genotyping assays were designed according to the target regions and tested through a genotyping reaction (Supplementary Fig. S2). Available assays were used for emulsion generation together with sheared genomic DNA extracted from blood and tissue samples. After PCR amplification and droplet detection on a Raindrop Sense droplet detector, the data were analyzed, and the fractions of mutant alleles were calculated, along with 95% binominal confidence intervals. For the lower bound of the confidence interval, 1/10000 was set as the threshold (Supplementary Fig. S3).

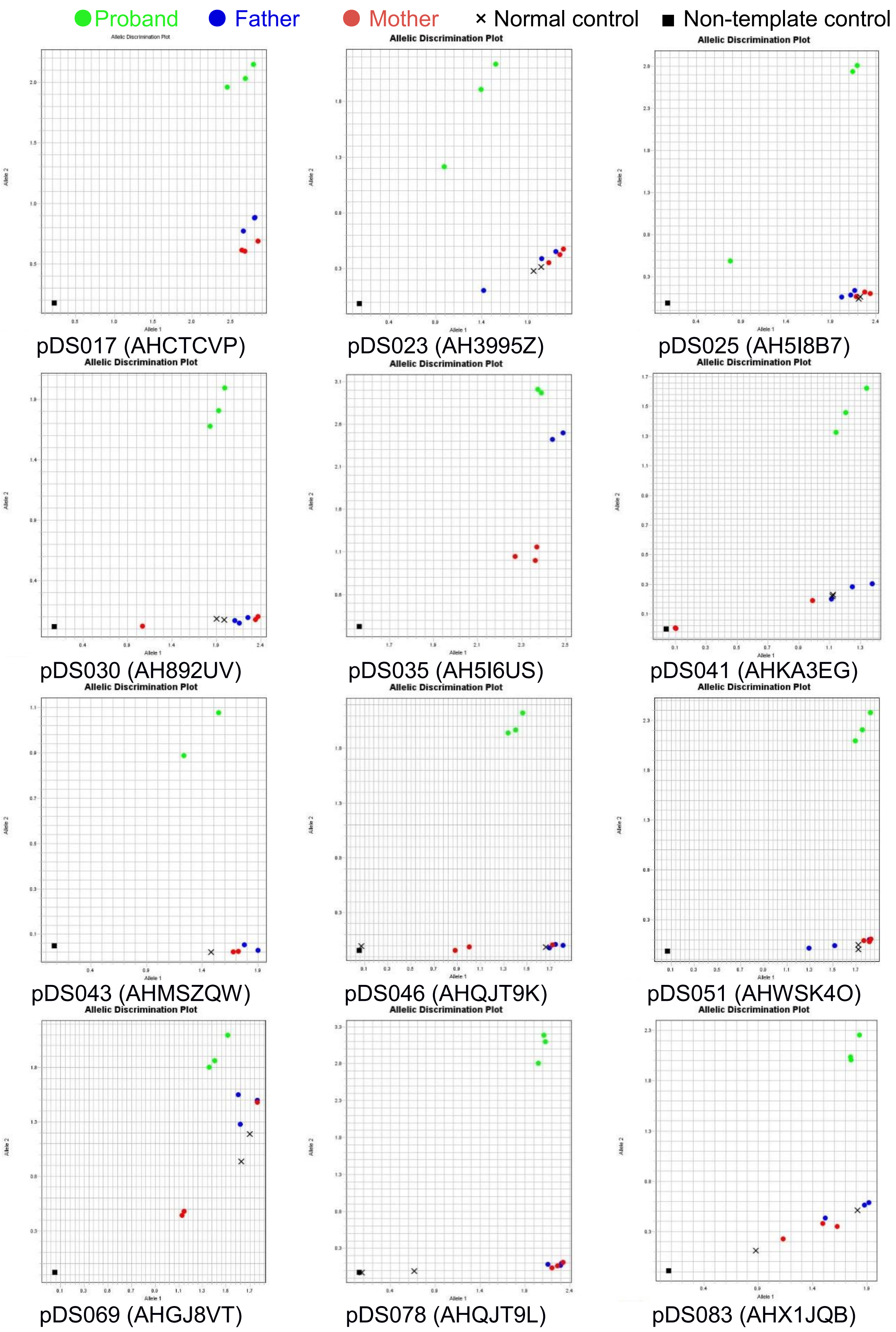
Proband
Father
Mother
Normal control
Non-template control
Allelic Discrimination Plot
Allele 1
Allele 2
pDS017 (AHCTCVP)
pDS023 (AH3995Z)
pDS025 (AH5I8B7)
pDS030 (AH892UV)
pDS035 (AH5I6US)
pDS041 (AHKA3EG)
pDS043 (AHMSZQW)
pDS046 (AHQJT9K)
pDS051 (AHWSK4O)
pDS069 (AHGJ8VT)
pDS078 (AHQJT9L)
pDS083 (AHX1JQB)

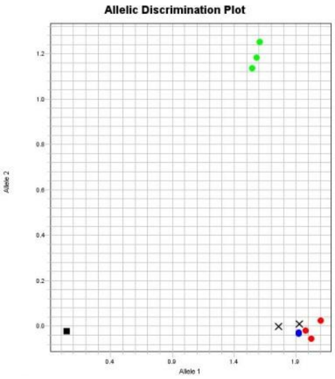

pDS087 (AHZAHG5)

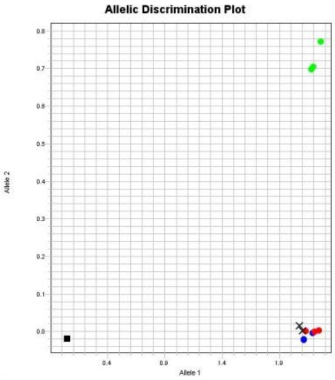

pDS093 (AH1SDTL)

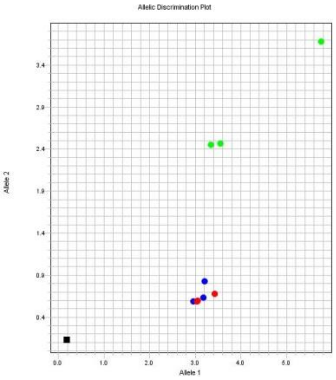
pDS094 (AH398OK)

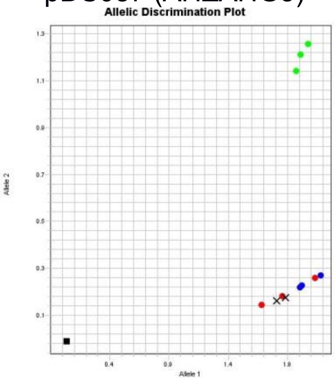

pDS095 (AH21BZT)

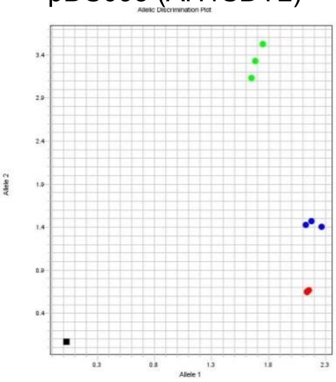
pDS101 (AH891DG)

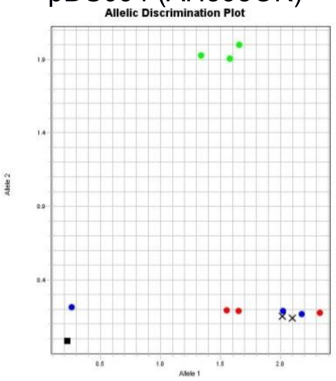

pDS106 (AHCTEC6)

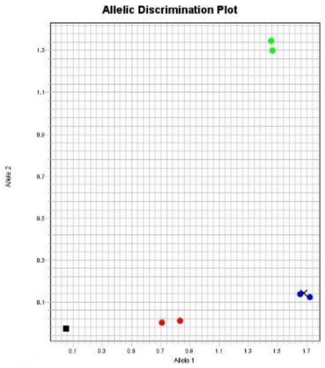

pDS117 (AH21CTR)

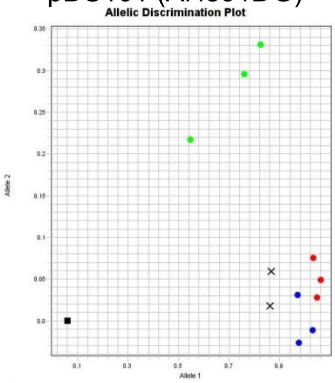

pDS120 (AHLJ0SZ)

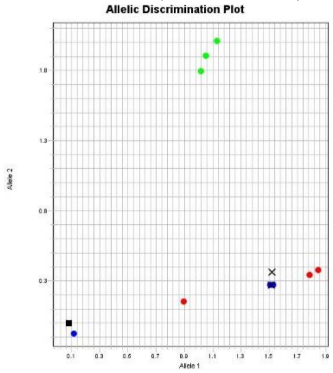

pDS123 (AHUAOSA)

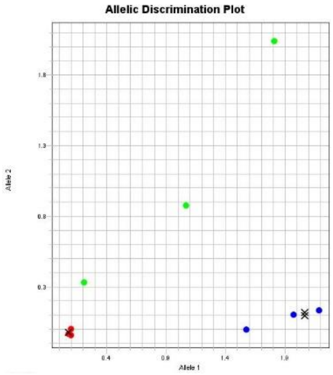

pDS124 (AH6R6IF)

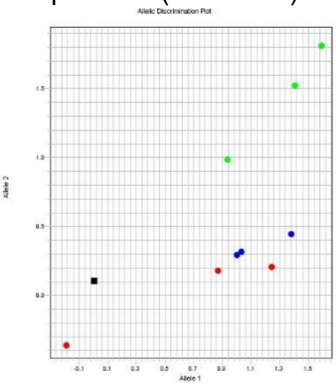
pDS125 (AH6R46V)

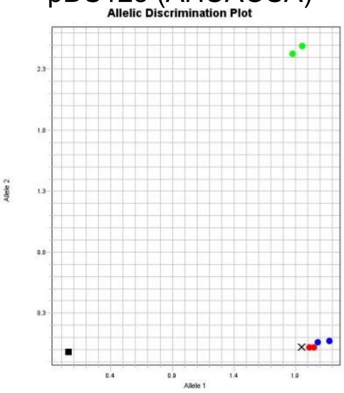

pDS126 (AH4AAZZ)

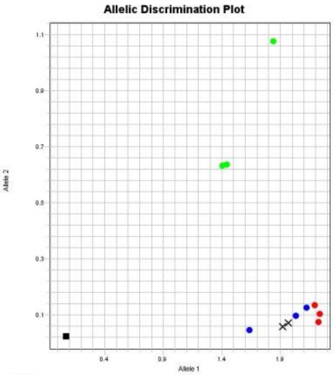

pDS127 (AHWSK4Q)

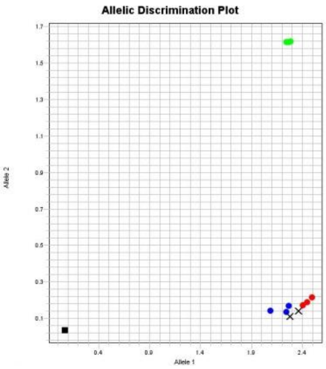

pDS134 (AH0JFNE)

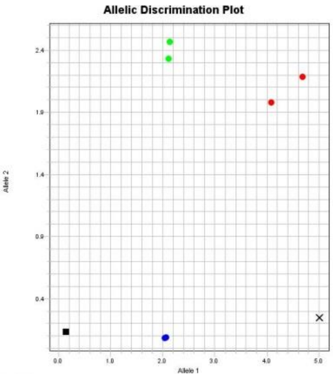

pDS136 (AHD2BCC)

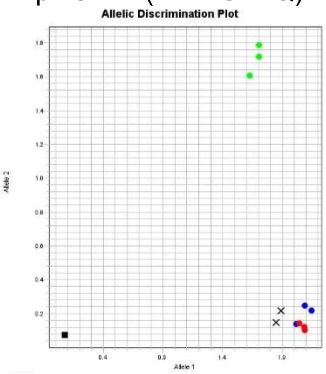

pDS141 (AH5I8CA)

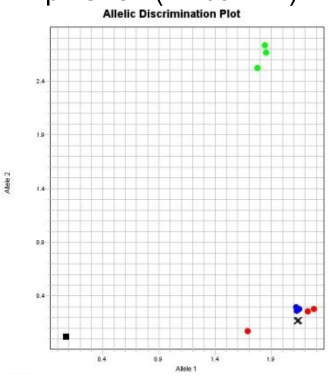

pDS149 (AHBKF6Z)

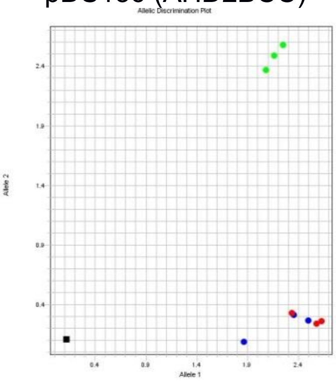

pDS157 (AHD2A1X)

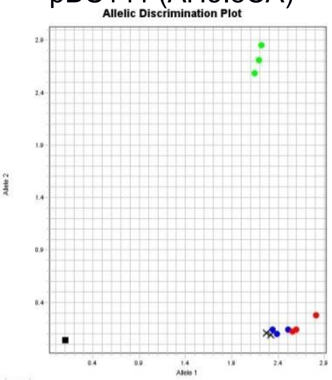

pDS158 (AHHS613)

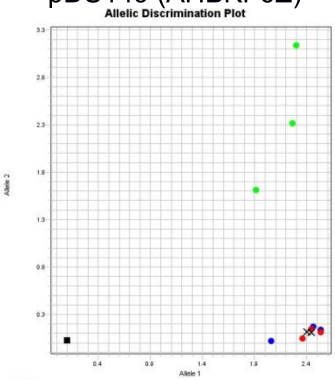

pDS159 (AHI148B)

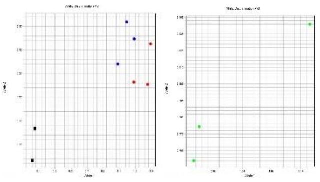
pDS164 (AH1SCB4)

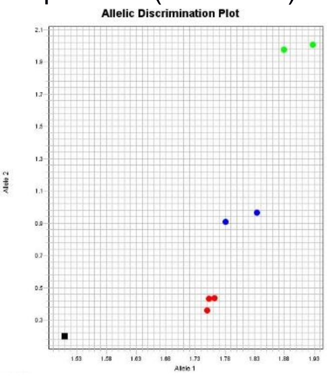

pDS166 (AHGJ7ED)

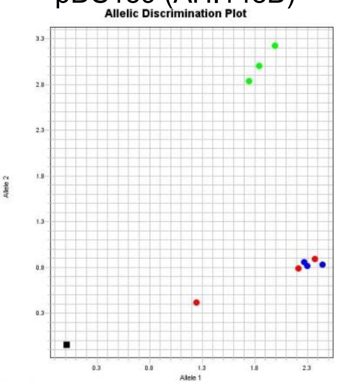

pDS179(AHFA875)

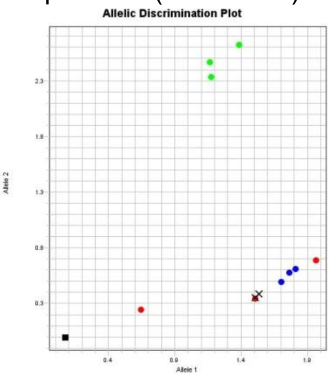

pDS191 (AHABH0S)

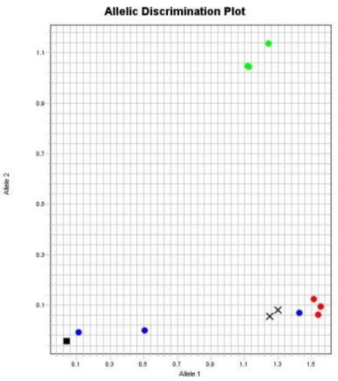

pDS208 (AHQJTHV)

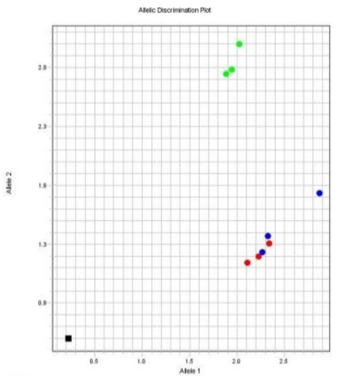

pDS210 (AH70268)

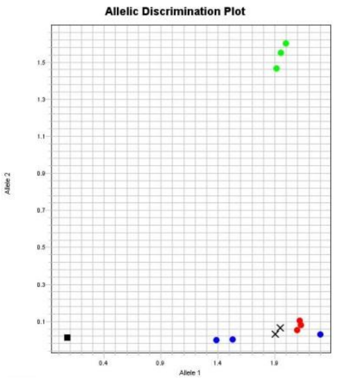

pDS276 (AHN1W5F)

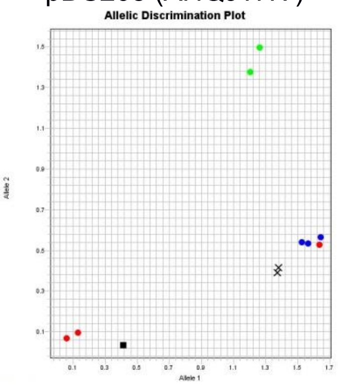

pDS277 (AHI15NN)

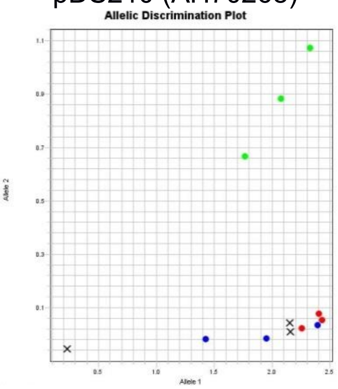

pDS278 (AHMSZQV)

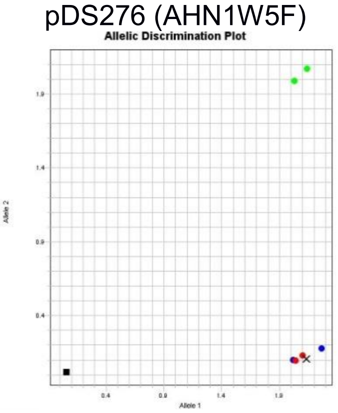

pDS282 (AHWSLJ3)

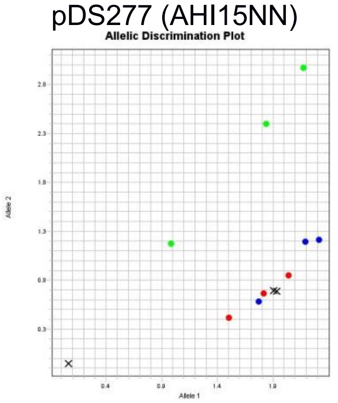

pDS283 (AHLJ1KN)

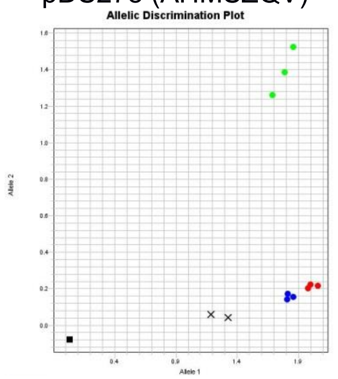

pDS284 (AHKA3TV)

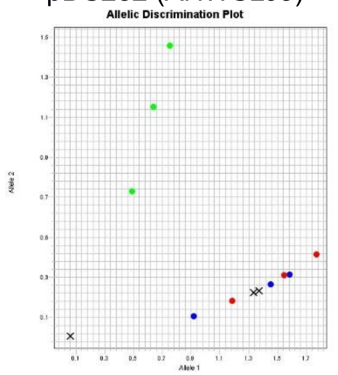

pDS285 (AHKA3EF)

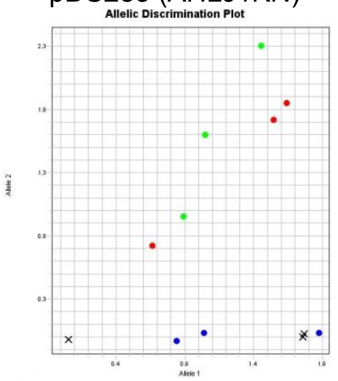

pDS287 (AHI1477)

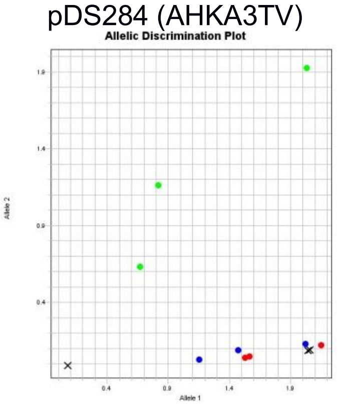

pDS289 (AHHS61Z)

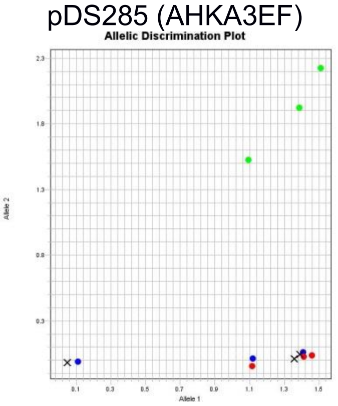

pDS290 (AHGJ8VR)

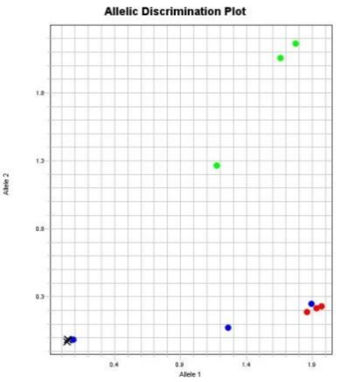

pDS291 (AHFBAPJ)

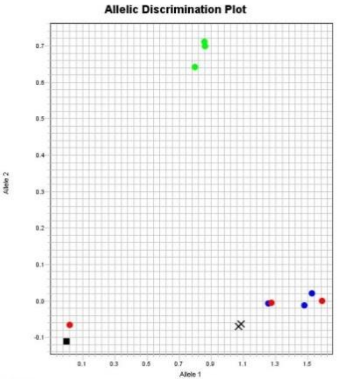

pDS294 (AHLJ1Z3)

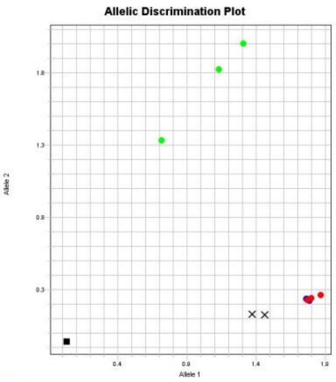

pDS297 (AHPAWIR)

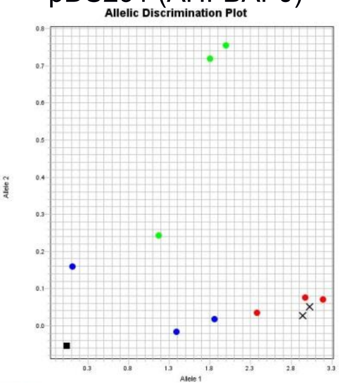

pDS299 (AHQJUOZ)

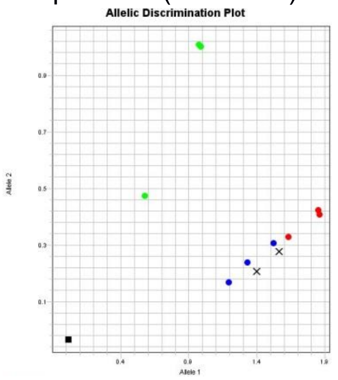

pDS300 (AHRSSU7)

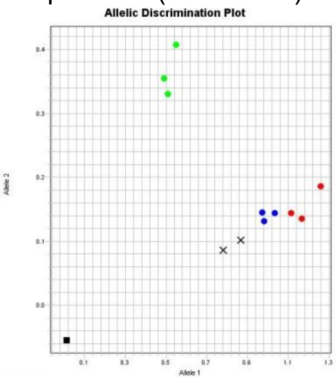

pDS301 (AHS1Q1F)

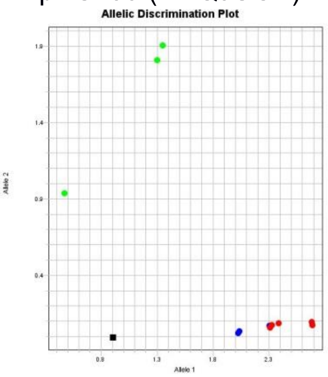

pDS302 (AHUAO7N)

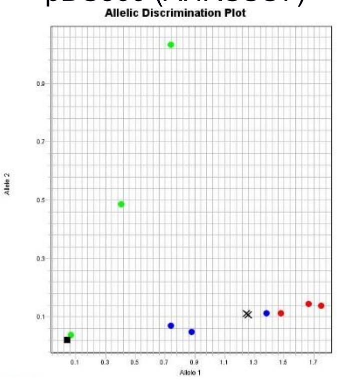

pDS303 (AHVJNDV)

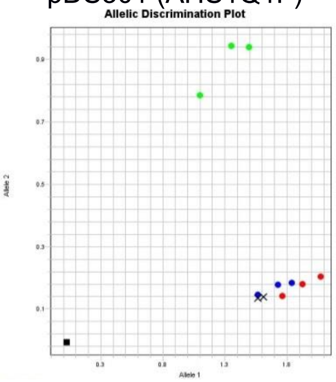

pDS305 (AHHS7HF)

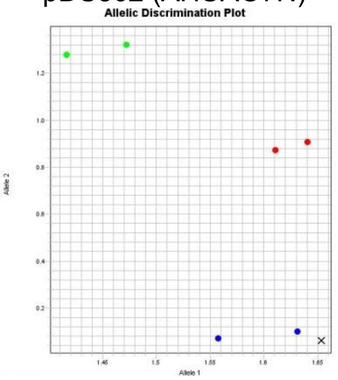

pDS307 (AHCTEY1)

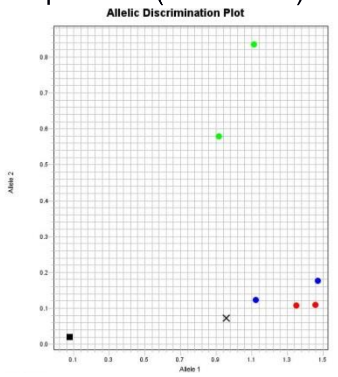

pDS310 (AH0JGI0)

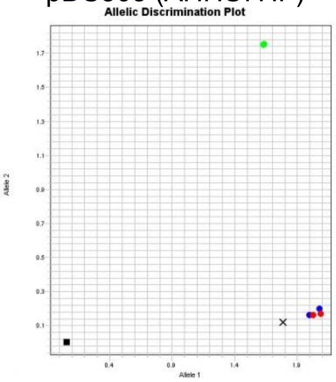

pDS311 (AH1SEO8)

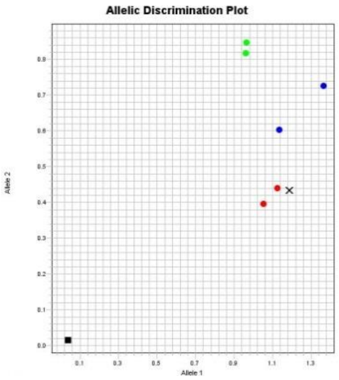

pDS312 (AH21CVG)

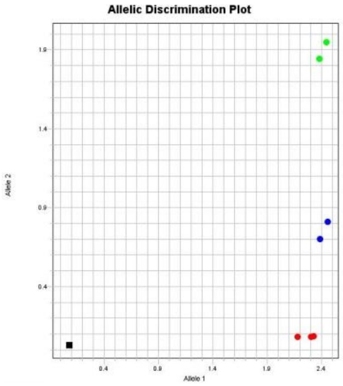

pDS314 (AH4AA1O)

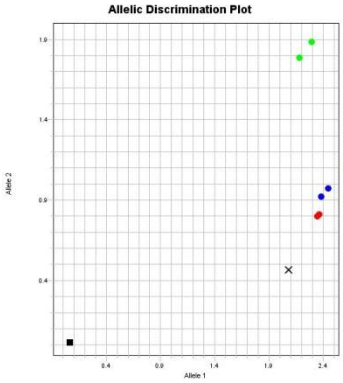

pDS315 (AHFBBLL)

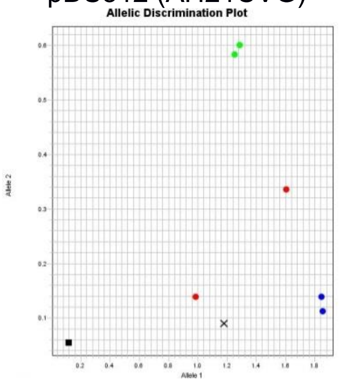

pDS316 (AHGJ9RT)

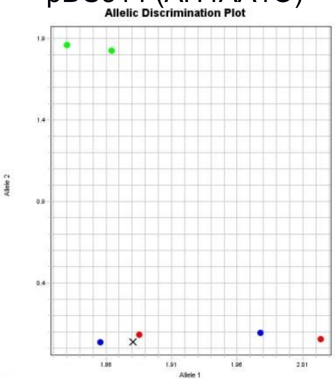

pDS318 (AHMS0RB)

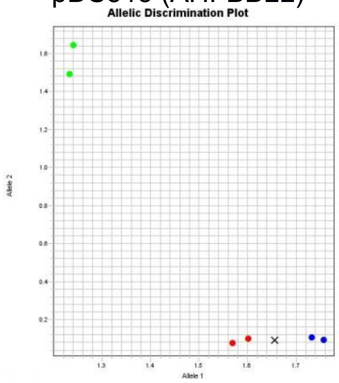

pDS319 (AHN1YXJ)

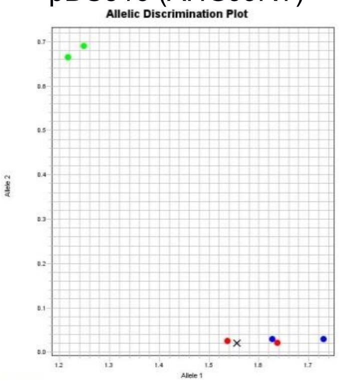

pDS320 (AHPAW3R)

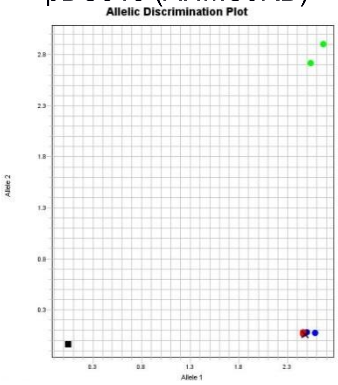

pDS321 (AHN1YY1)

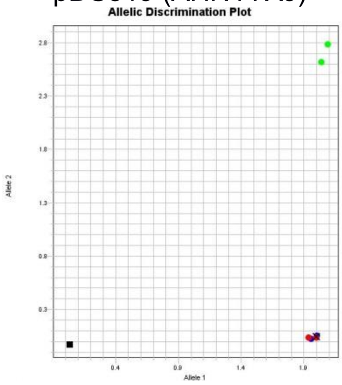

pDS322 (AHPAW49)

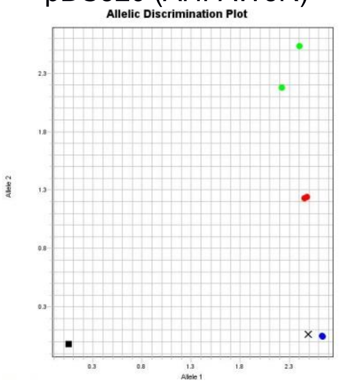

pDS323 (AHQJVBH)

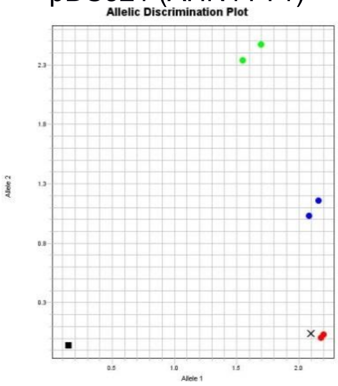

pDS324 (AHRSTHP)

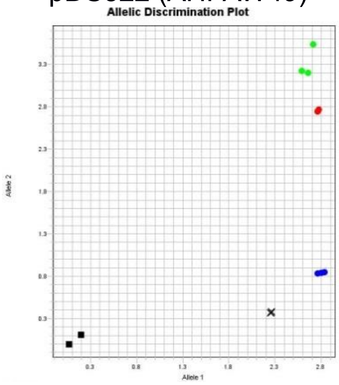

pDS327 (AHX1KMH)

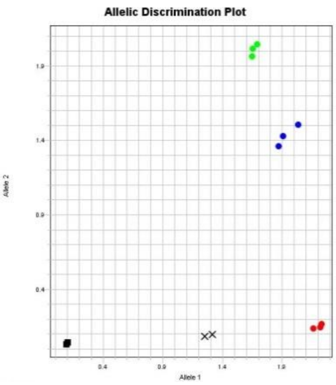

pDS328 (AHZAISP)

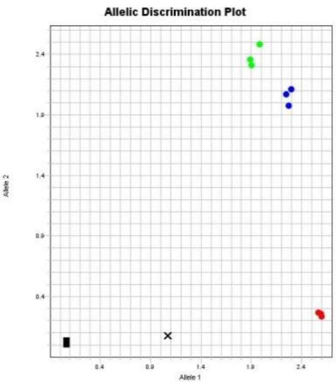

pDS329 (AH0JGYX)

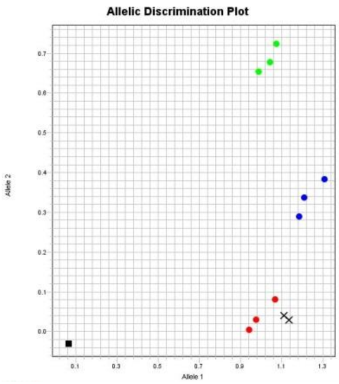

pDS003 (AH1SFB3)

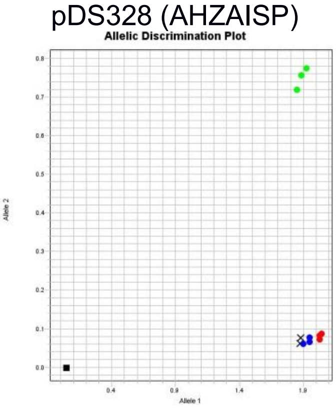

pDS330 (AHLJ22T)

Figure S3. TaqMan end-point genotyping reaction results and the related assay IDs. The manufacturer's assay IDs are provided under each genotyping phase chart. All assays were designed under part number 4331349. In the genotyping reaction phase charts, each data point represents an independent genotyping reaction result. The coordinates on the x-axis and y-axis stand for log-transformed relative signal intensities of the two fluorescent dyes. The suspected heterozygous mutation carrier probands are shown as green dots, their fathers are shown as blue dots, and their mothers are shown as red dots. Black crosses stand for normal controls, and black boxes stand for non-template controls (NTCs). If a set of TaqMan primers can distinguish the wild type and mutant alleles, signals from both channels should be detected from the positive controls (probands), and signals from only the wild type allele should be detected from the negative controls and parents, resulting in green dots in the top right corner of the graph, black crosses at the bottom of the graph, and either/both blue and red dots at the bottom. Otherwise, the assays are determined to have difficulty distinguishing the wild type and mutant alleles.

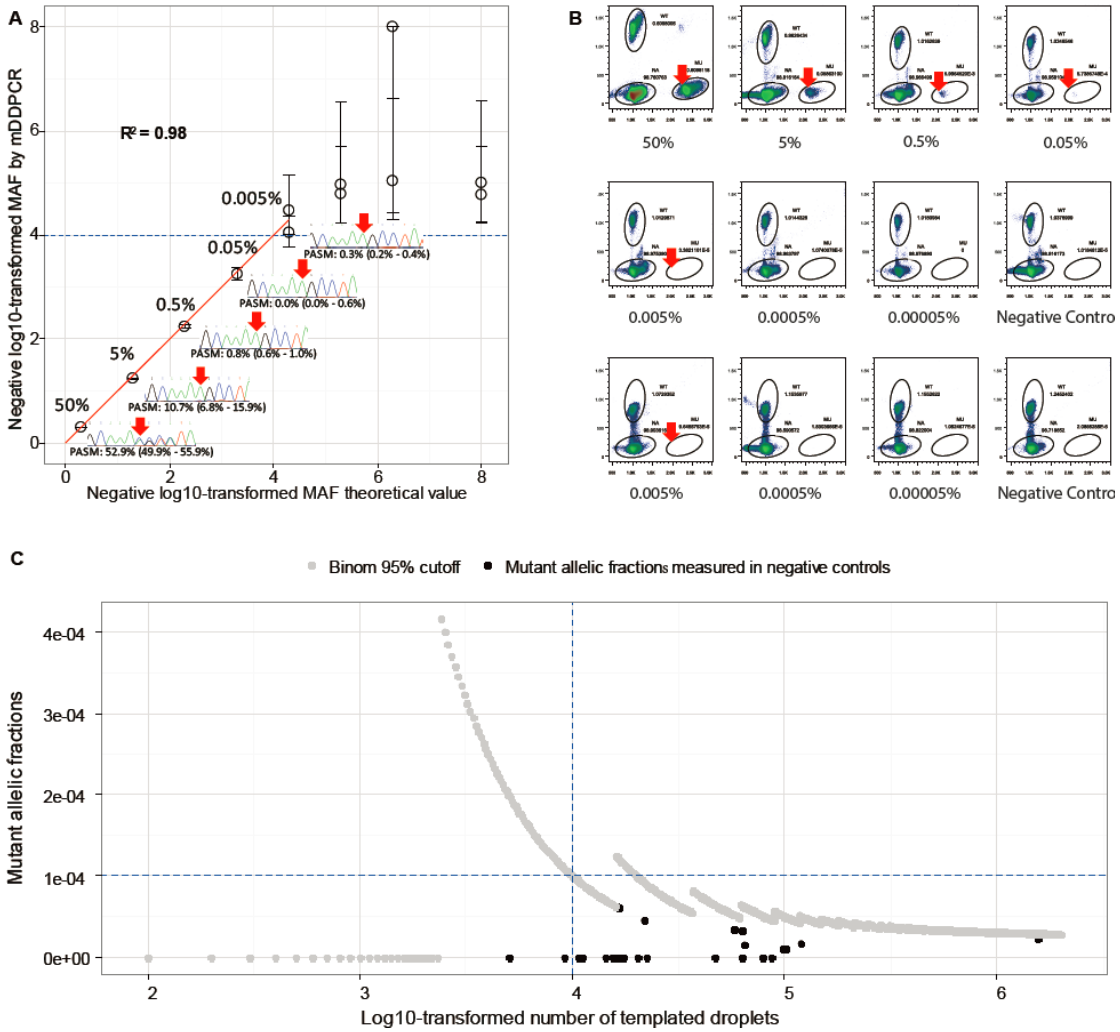

Figure S4. Benchmarking with serial dilution standards, negative controls and similarity correction.

(A) Serial dilution standards were tested using PCR with Sanger sequencing, PASM and mDDPCR. The x-axis shows the theoretical MAFs (log10 transformed) for each sample point. The y-axis shows the mDDPCR results (log10 transformed) and the 95% binomial confidence intervals (CIs). Sanger chromatograms for the same standards are also provided beside each standard point. The PASM maximum estimators and 95% credible intervals are provided under each Sanger chromatogram. Sanger sequencing could not efficiently distinguish mutant alleles for standards under 5%. PASM could detect rare mosaicism cases with MAFs of approximately 0.5%. For serial standards with MAFs higher than 0.005%, the log10 transformed theoretical MAFs were highly corrected with the mDDPCR results (R^2 = 0.98). The performance of mDDPCR for MAFs lower than 0.005% was similar to the negative controls. (B) mDDPCR flow cytometry scatter plots for serial dilution standards. For MAFs higher than 0.005%, signal clusters of the mutant allele could be visualized with gradient shrinkage along with MAF theoretical values. For theoretical MAFs at 0.005%, very weak signal clusters for mutant alleles could be observed. For theoretical MAFs lower than 0.005%, signal clusters could only be visualized for wild type signals. (C) Evaluation of mDDPCR performance on negative controls. Different TaqMan assays were tested on negative control samples, and the log10 transformed data points are shown as black points. Empirical error rates are therefore estimated to be $2.2*10^{-5}$. Using this error rate for the null model, upper bound 95% binomial CIs were estimated for different numbers of templated droplets (WT + MU); the boundary is plotted in gray. Hence, 0.01% is used as the cutoff for the 95% CI lower bound for mDDPCR analysis (blue horizontal line) for samples with total templated droplets over 10^4.

A

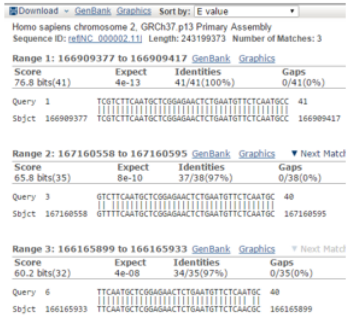

Part of exon 5

B

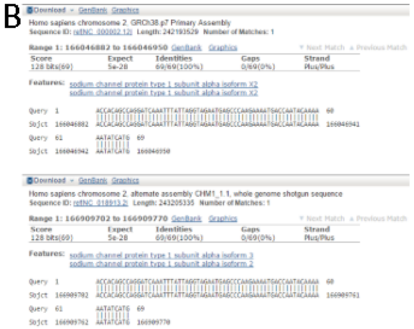

Part of exon 15

C

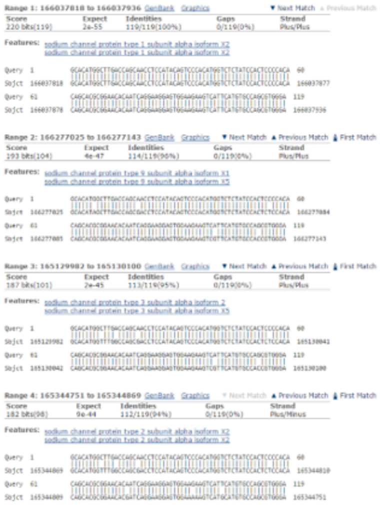

Part of exon 9

D

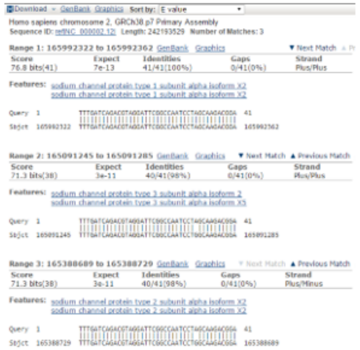

Part of exon 26

E

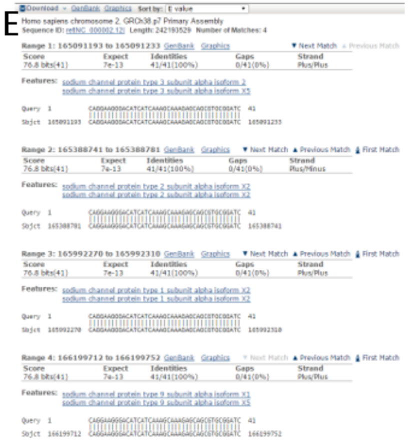

Part of exon 26

Figure S5. Genomic regions showing high similarity to *SCN1A* genes.

The highly similar regions are clustered in particular regions of the *SCN1A* sequence, according to the reference genomes hg19 and GRCh38. Heterozygous probands would be found to have an MAF of 1/6 if there are two similar regions (A and D), 1/4 if there is one similar genomic region (B), and 1/8 if there are three similar regions (C and E). A correlation could be found with the help of the BLAST and BLAT search tools.

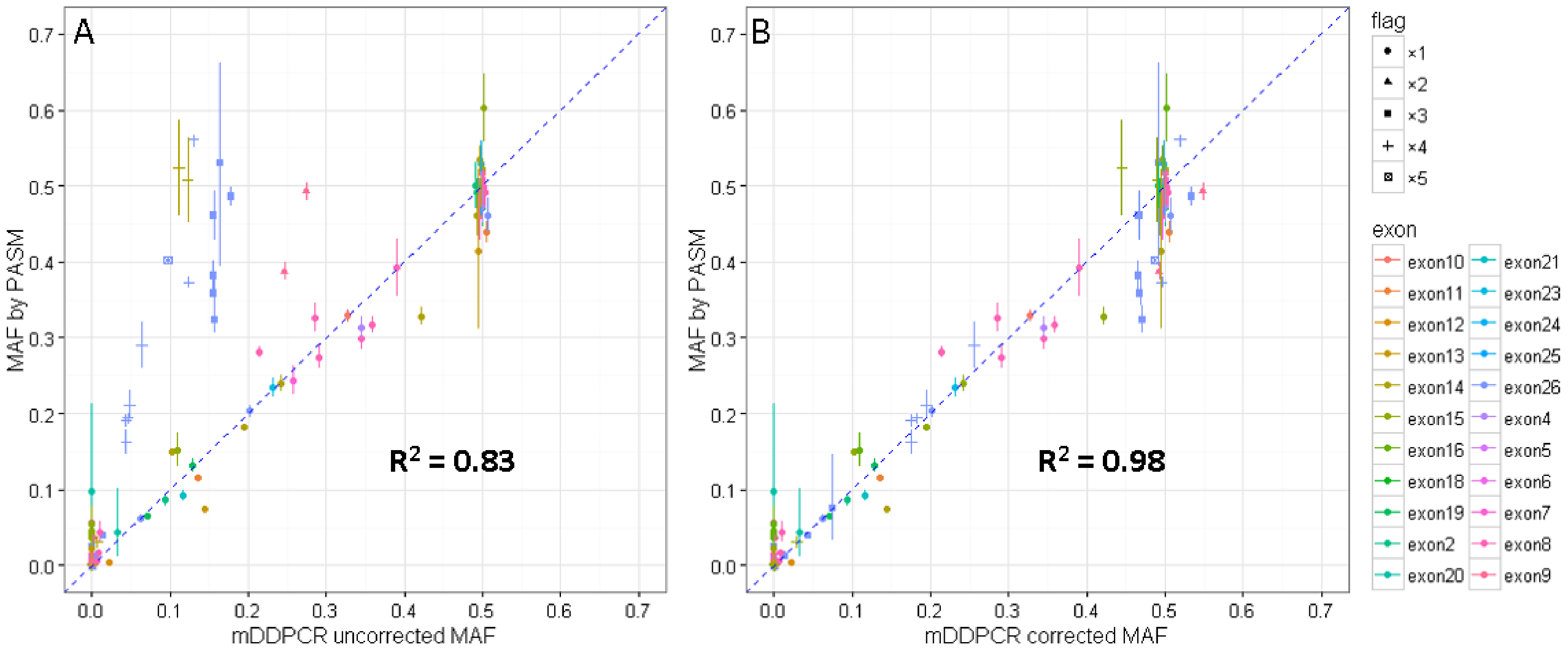

Figure S6. MAFs measured by mDDPCR before and after correction for similar regions. Correlations with PASM are shown. (A) Before correction for similar regions, data from mDDPCR show a weak correlation with the PASM validation results (R^2 = 0.83). The x-axis shows the log-transformed MAFs, and the y-axis shows the PASM MAF estimations with 95% CIs. Biased data are clustered in particular around exons 9, 15 and 26, and a linear transformation could be carried out to compensate for the skewed data points. (B) After correction for similar genomic regions using BLAT and BLAST, the MAFs measured by mDDPCR are highly correlated with the MAFs measured by PASM (R^2 = 0.98, p-value < 2.2e-16 by an F test, degrees of freedom = 170). The colors show different exons of *SCN1A*, and the shapes stand for similar genomic regions.

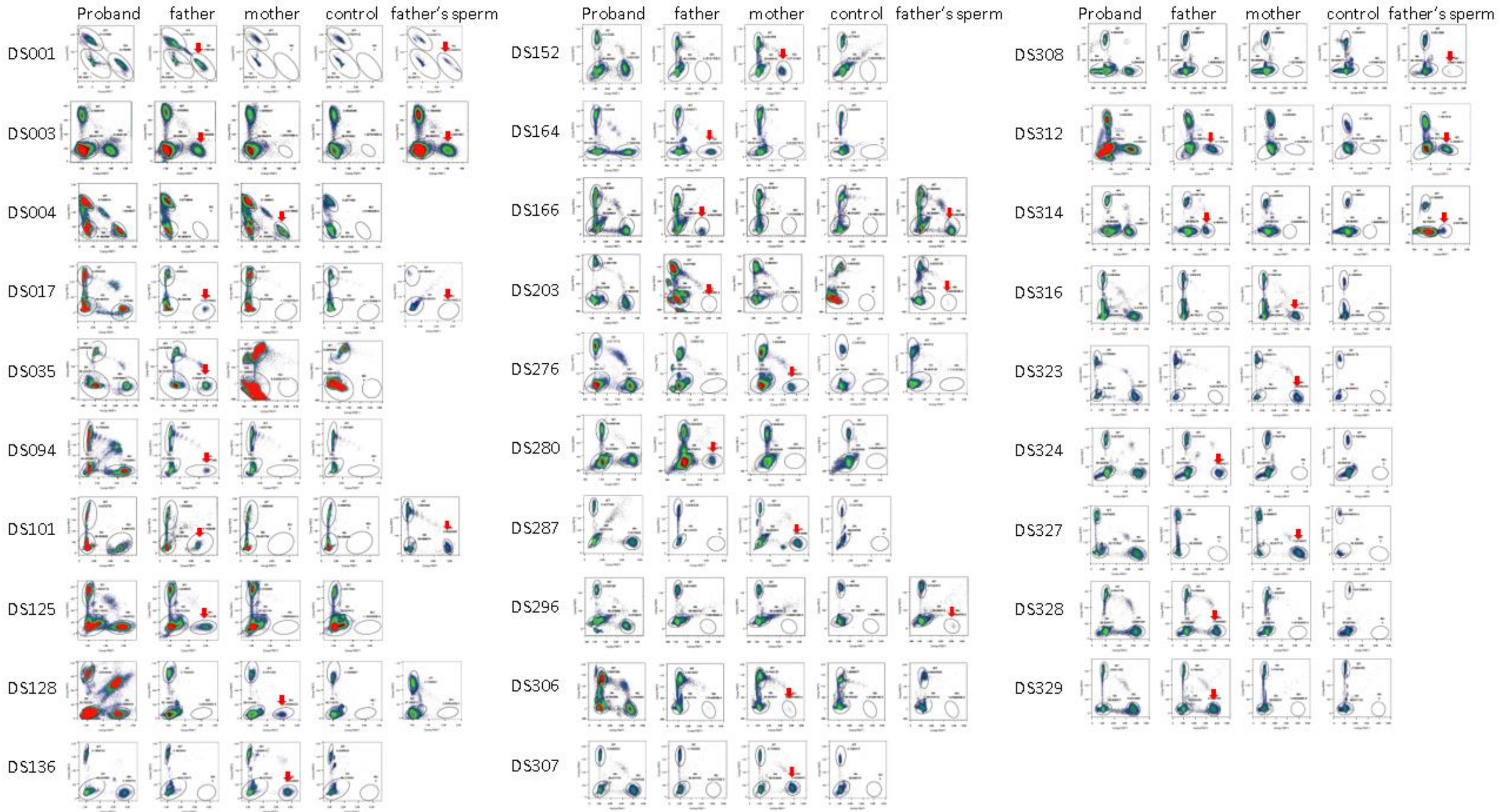

Figure S7. Raw flow chromatography clusters of mDDPCR analysis for the positive mosaic families.

For each family, the proband data were first provided. Positive clusters for wild type (WT) and mutant (MU) are shown, and both clusters have a similar number of data points, reflecting similar molecules of wild type and mutant alleles. The MAFs in the probands are typically near 50%. Blood samples from the mosaic parents and non-mosaic parents are shown before negative controls. Paternal sperm samples are also provided to help evaluate the hypothesis that sperm should have higher mutation allele frequencies.

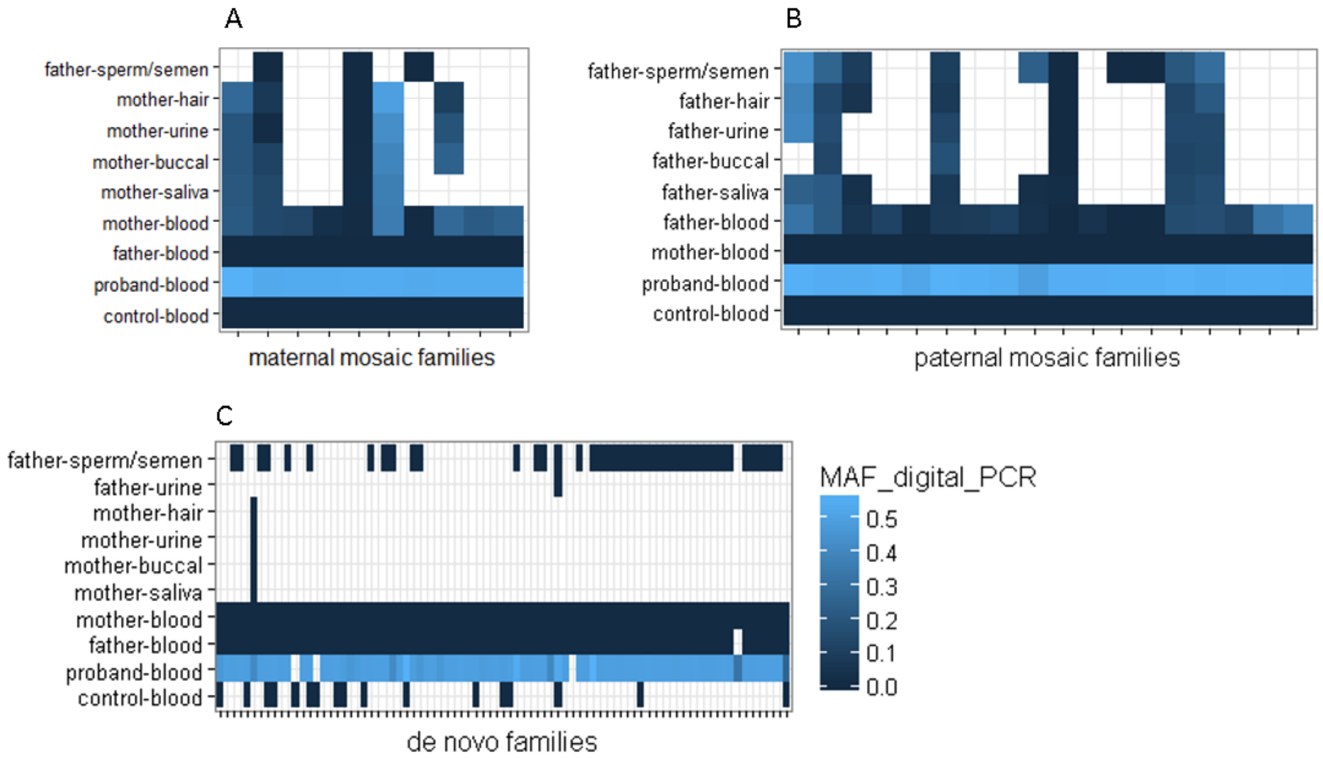

Figure S8. Multiple peripheral samples collected in all families.

The samples included parental hair follicles, urine, buccal epithelium samples, saliva, and paternal purified sperm/semen samples. mDDPCR-measured MAFs in the probands and control blood samples are also shown. The different colors represent different MAFs detected by mDDPCR in all samples. (A) Multiple samples from maternal mosaic families. (B) Multiple samples from paternal mosaic families. Multiple samples from the same mosaic parent are detected with different MAFs. Most MAFs from different tissues resemble the blood MAF. However, paternal sperm and parental hair MAFs showed the greatest differences. (C) Multiple samples from families considered to be *de novo* families based on mDDPCR. Paternal sperm samples and parental tissue samples show similar MAFs (approximately 0%) compared to parents and negative controls. However, in some of the *de novo* families, the probands show MAFs that indicate mosaicism.

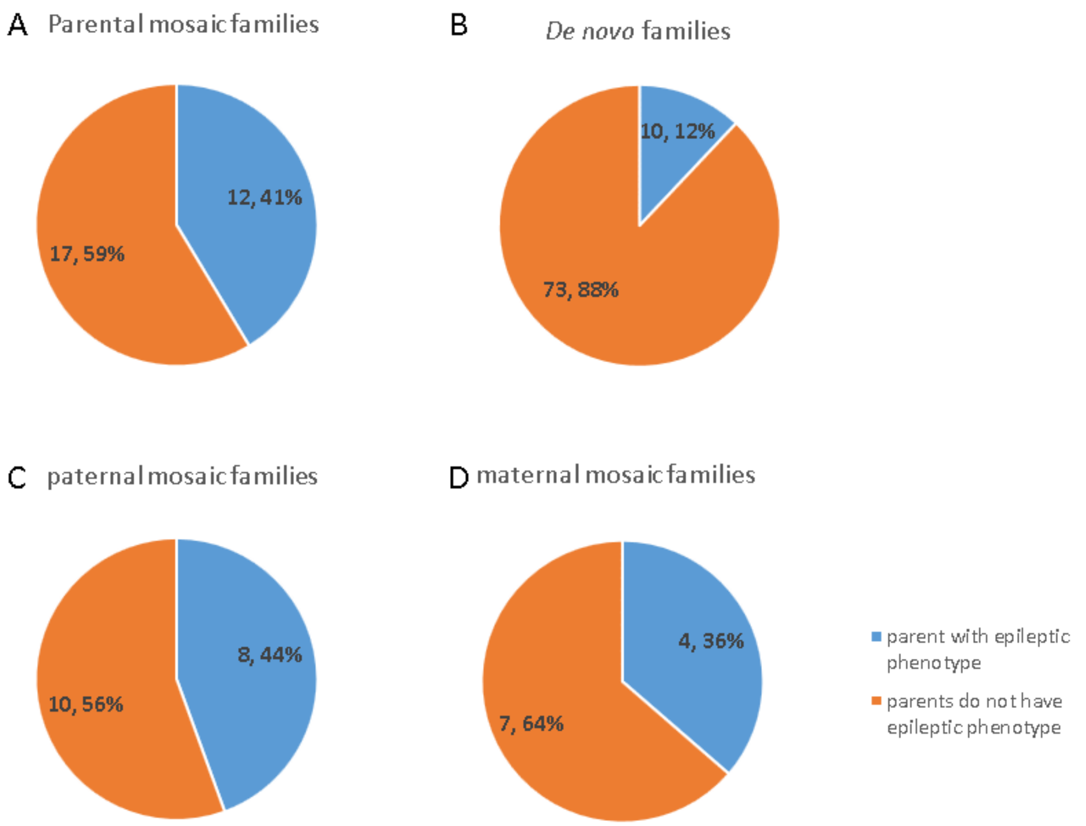

Figure S9. Parental epileptic phenotype profile.

(A) Familial epileptic phenotypes are summarized in the pie charts. DS families in which the parents had epilepsy are shown in blue. Families in which the parents did not have epilepsy are shown in orange. (B) Parental phenotype in *de novo* families. Paternal (C) and maternal (D) mosaic families are independently summarized, and no phenotypic parent-of-origin bias is found (odds ratio = 1.4, p-value = 0.72 by a two-tailed Fisher's exact test). The proportion of first degree relatives having had an epileptic phenotype in mosaic families is significantly higher than that in *de novo* families (odds ratio = 5.1, p-value = 0.0019 by a two-tailed Fisher's exact test, A and B).

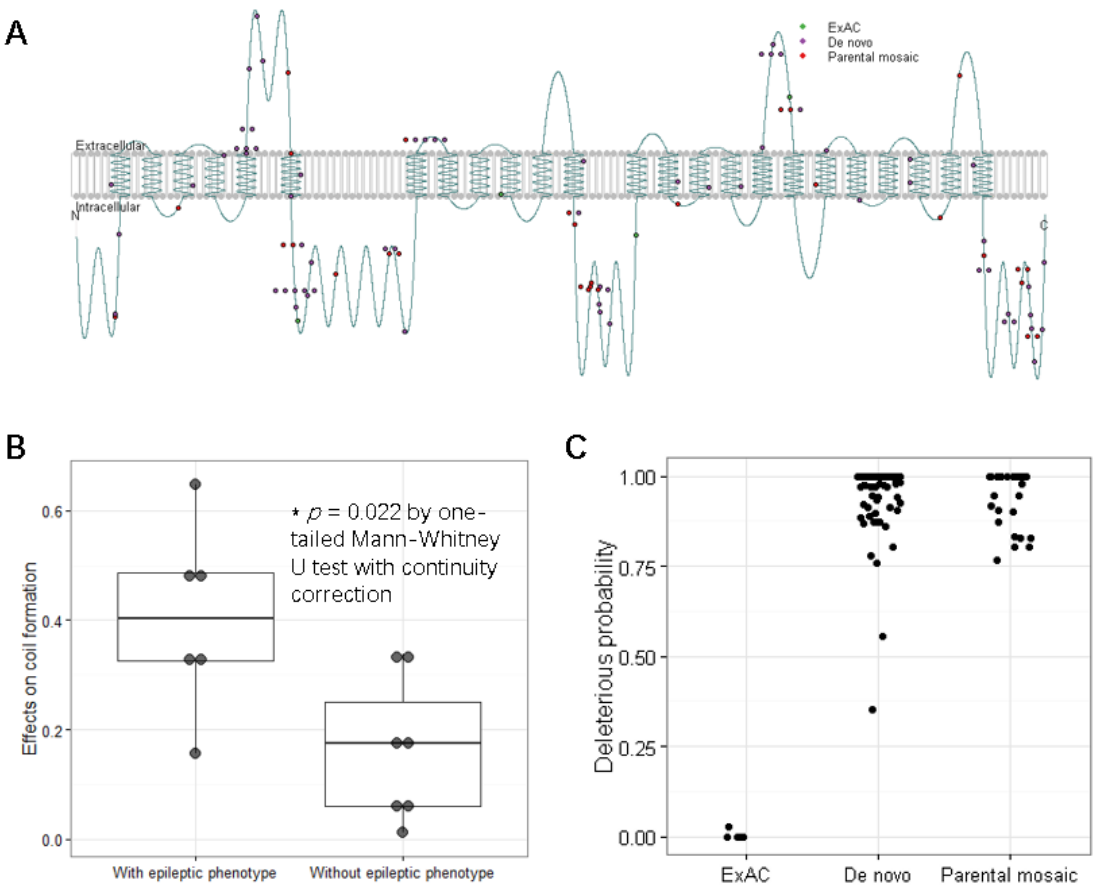

Figure S10. Functional predictions for parental mosaic mutations and *de novo* mutations. SNPs in the coding regions of *SCN1A* with population frequencies over 1% were selected from ExAC and served as negative controls. (A) The distribution of mutations detected in the SCN1A protein in the DS affected families. In the transmembrane alpha-helix region, only one parental mosaic variant was observed. Other parental polymorphisms were found in the intracellular or extracellular coil regions. (B) Further exploration of the functional effects of coil formation in mosaic parents showed that variants found in mosaic parents with an epileptic phenotype had significantly larger effects on coil formation (p-value = 0.022 by a Mann-Whitney U test with continuity correction) than those in mosaic parents who do not have epileptic phenotypes. (C) *SCN1A* mutations from the DS families were used to determine the probabilities of deleterious effects for missense variants using iFish. The probabilities for nonsense and frameshift variants were set to 1, and the probabilities for synonymous mutations were set to 0. Variants in *de novo* families and parental mosaic families had similar deleterious probabilities that were much higher than those of common SNPs from ExAC.

Supplementary Tables:

Table S1: New potentially pathogenic *SCN1A* variants found in DS probands, detected by Sanger sequencing and validated by qPCR end-point genotyping as well as mDDPCR

Family	Mutation	Origin
DS275	NM_001165963.1:c.1092delC	*de novo*
DS277	NM_001165963.1:c.5713delG	*de novo*
DS282	NM_001165963.1:c.5170G>A	*de novo*
DS283	NM_001165963.1:c.3372delT	*de novo*
DS284	NM_001165963.1:c.337C>A	*de novo*
DS285	NM_001165963.1:c.505T>C	*de novo*
DS286	NM_001165963.1:c.2877_2903delTATGGAGGTTGCTGGTCAAGCCATGTG	*de novo*
DS287	NM_001165963.1:c.1462_1466delAAGTT	maternal
DS289	NM_001165963.1:c.2936G>A	*de novo*
DS290	NM_001165963.1:c.3434_3435delTG	*de novo*
DS291	NM_001165963.1:c.4549A>T	*de novo*
DS294	NM_001165963.1:c.1077T>A	*de novo*
DS295	NM_001165963.1:c.3327_3328delCA	*de novo*
DS298	NM_001165963.1:c.4803delC	*de novo*
DS299	NM_001165963.1:c.4995_4996insATT	*de novo*
DS300	NM_001165963.1:c.4581+2T>A	*de novo*
DS303	NM_001165963.1:c.5513C>T	*de novo*
DS304	NM_001165963.1:c.5174G>T	*de novo*
DS305	NM_001165963.1:c.5024T>C	*de novo*
DS307	NM_001165963.1:c.971A>C	maternal
DS309	NM_001165963.1:c.1934_1935delTG	*de novo*
DS310	NM_001165963.1:c.4484delG	*de novo*
DS311	NM_001165963.1:c.780dupA	*de novo*
DS312	NM_001165963.1:c.1850_1851delGA	paternal
DS313	NM_001165963.1:c.4476G>T	*de novo*
DS316	NM_001165963.1:c.4921G>A	maternal
DS318	NM_001165963.1:c.3646G>T	*de novo*
DS319	NM_001165964.1:c.543delA	*de novo*
DS323	NM_001165963.1:c.4339-1G>A	maternal
DS324	NM_001165963.1:c.3327_3328delCA	paternal
DS325	NM_001165963.1:c.3995G>A	*de novo*
DS329	NM_001165963.1:c.488G>A	paternal
DS330	NM_001165963.1:c. 3547delG	*de novo*

Table S2: STR primers and their genomic positions in the *SCN1A* locus

Locus	position start[a]	position end[a]	Left primer	Right primer	Distance
AEMa081we1	chr2:164490522	chr2:164490761	AACCTGGGAAACTGGTTTTTAT	TCATGTGCTTTTCATTCCAA	124 bps
D2S2157	chr2:165827178	chr2:165827468	TTCCCCAAAAGGCACCAA	GTAGCAAAATCCAAACCAAACCGTA	136-154 bps
D2S124	chr2:166145063	chr2:166145360	TCAACTTTTTGATTGTTACTTGC	CCCCATTATGGAAATTGTTAG	157-163 bps
D2S2363	chr2:166557995	chr2:166558364	TACATAAAGCCCCTGTCA	GTCATCAATTCTAGTCCCAA	163-211 bps
SCN1A	chr2:166845670	chr2:167005642	-	-	-
D2S1395	chr2:167871791	chr2:167872127	ATCCCAAACACGATGTTGAT	GGCATGAAAGGAAACCTACA	119 bps
D2S1379	chr2:167871781	chr2:167872153	ATCCCAAACACGATGTTGAT	TCAAACTCAATTCCTTCATCTG	148 bps

[a:]Genomic positions based on hg19/GRCh37

Table S3: STR analysis for mosaic candidate families

Run	Sample	AFMA081WE1	D2S124	D2S2363	D2S2157	D2S1379	D2S1395
D2S124		120.7	160(M)	190.2	144.6	151.7	117.3
D2S2157		122.7	161.9(F)	205.9	144.6	151.7	121.2
D2S1395		120.7	155.9	190.2	134.3	151.9	117.5
		122.7	161.6	205.8	144.7	151.9	121.2
		120.6	159.9	190.2	144.6	146.4	116.3
		122.6	161.8	205.9	144.6	151.8	121.3
D2S1379		118.7(F)	155.9	201.8(F)	134.2	151.7	121.3
D2S1395		122.6(M)	161.6	205.8(M)	144.6	151.7	121.3
		118.8	155.9	201.9	134.3	151.8	121.2
		122.7	161.6	205.9	144.7	156	125.3
		122.6	155.9	205.8	134.3	151.8	121.2

		122.6	161.5	205.8	144.7	155.9	125.2
AFMA081WE1		118.8(M)	155.9(F)	201.8(M)	142.6?	147.4	117.2
D2S124		122.6(F)	159.8(M)	205.8(F)	144.7	151.7	121.1
D2S2363		120.7	156	205.8	142.4	147.3	113.9
D2S1395		122.7	159.9	190.1	144.6	147.3	117.2
		118.9	159.9	201.7	142.5	151.8	121.2
		118.9	159.9	207.7	144.7	151.8	121.2
D2S2363		120.8	155.9	199.9(F)	134.3	146.4	116.4
D2S2157		122.7	161.7	201.9(M)	148.9	156.1	125.4
D2S1379		120.7?	157.3	190.2	134.2	146.3	116.2
D2S1395		122.6	161	199.8	144.6	155.9	125.4
		118.8	155.9	190	134.1	147.3	121.1
		122.7	157.4	201.7	148.9	147.3	125.3
AFMA081WE1		118.9(M）	161.6	190.2(F)	134.3	146.4	116.3
D2S2157		122.8(F)	161.6	205.9(M)	144.7	160.1	129.5
D2S1379		120.7	161.7	190	138.2	151.9	121.2
D2S1395		122.6	161.7	190	144.7	160	129.4
		118.7	157.7	205.8	134.2	146.4	116.2
		120.6	163.1	205.8	146.8	151.8	121.2
		122.7	155.9(M)	190.3(F)	138.2	147.4	117.3
		122.7	161.7(F)	205.9(M)	144.7	150.9	120.5
		121.1	161.7	190.2	144.7	150.9	120.3
		121.1	161.7	190.2	144.7	150.9	120.3
		120.8	156	199.8	139	147.3	117.3
		122.7	161.7	205.8	145	151.8	121.3
AFMA081WE1		118.9(F)	161.7(F)	203.9(F)	134.3	141.9	112.3
D2S2363		126.5(M)	163.6(M)	190.3(M)	136.3	156	125.4
D2S2157		118.7	161.8	203.9	136.3	141.8	112.2

D2S1379		122.5	161.8	205.9	146.9	156	125.3
D2S1395		122.7	156	190.2	134.2	155.9	125.3
		126.5	163.6	190.2	134.2	155.9	125.3
D2S124		124.5(F)	160(F)	203.9(F)	139	155.8	125.2
AFMA081WE1		122.6(M)	156.1(M)	205.9(M)	145	155.8	125.2
D2S2363		124.5	160	203.8	144.9	155.8	125.2
		122.7	162	207.8	144.9	155.8	125.2
		122.6	153.5	190.1	138.3	156	125.4
		122.6	160.3	205.8	144.7	156	125.4
D2S2363		118.7(F)	156(F)	205.8(F)	144.7	147.5	116.2
D2S1395		122.6(M)	160(M)	190.2(M)	144.7	161	125.3
		118.8(F)	155.9	190.2	144.7	146.4	116.2
		118.8	155.9	205.8	149	159.2	129.4
		118.8	156	190.2	144.5	151.7	121.2
		122.6	160	207.8	144.5	155.9	125.4
AFMA081WE1		120.6(F)	159.8(F)	205.8(F)	142.5	151.8	121.1
D2S124		118.7(M)	157.8(M)	190.1(M)	144.7	151.8	121.1
D2S2363		120.7	159.9	190.1	142.5	151.8	121.2
		122.6	161.8	205.8	144.7	151.8	121.2
		118.6	157.6	190.2	142.5	147.4	117.1
		122.5	161.5	190.2	144.6	151.8	121.2
		122.6	161.5	190.1	134.2	151.8	116.2
D2S124		122.6	161.5	190.1	148.9	146.3	121.1
D2S2363		122.6	155.8	190.1	134.2	146.3	116.2
D2S2157		122.6	161.6	205.8	144.6	151.8	121.1
		118.6	159.9	190.2	134.1	151.7	121.2
		122.5	161.8	207.8	148.9	151.7	121.2
AFMA081WE1		120.5(M)	159.9(M)	190.2(F)	134.3	147.4	117.1

D2S124		122.5(F)	161.8(F)	205.9(M)	144.7	156	125.3
D2S2363		118.8	156.9	190.1	134	147.3	117.2
D2S2157		122.6	161.6	201.8	146.6		125.3
D2S1379		120.7	156.1	199.8	144.7	146.3	116.1
D2S1395		122.6	160	205.8	144.7	155.9	125.1
D2S124		122.7	161.9(F)	205.8(F)	144.7	155.9	125.2
D2S2363		122.7	159.9(M)	207.8(M)	144.7	155.9	125.2
D2S1379		122.7	159.9	203.7	144.7	150.9	120.2
D2S1395		126.5	161.9	205.7	144.7	156	125.3
		122.6	156	192	144.6	146.4	116.1
		122.6	160	207.7	144.6	156	125.2
AFMA081WE1		118.9(M)	155.9	190.2	144.7	155.9	125.2
D2S1379		120.8(F)	161.6	190.2	146.8	155.9	125.2
D2S2363		120.7	145.4	190.2	144.6	151.7	121.2
D2S1395		122.6	149.9	205.8	144.6	155.8	125.2
		118.8	161.5	190.1	144.7	151.8	121.2
		120.7	161.5	190.1	146.8	156	125.2
AFMA081WE1		122.7	154.6	192.2(F)	144.7	151.9	121.3
D2S124		122.7	159.9	205.9(M)	146.8	156.1	125.4
D2S2363		122.6	157.9	190.2	144.7	151.8	121.2
		122.6	159.9	192.1	146.8	155.9	125.3
		118.7	154.4	201.9	144.5	151.8	121.2
		122.6	159.9	205.9	144.5	156	125.3
AFMA081WE1		122.6(F)	155.9(F)	190	144.6	145.3	125.2
D2S2363		120.7(M)	161.7(M)	190	146.7	150	129.3
D2S1395		120.7	156	190.2	138.2	147.2	117.2
D2S1379		122.6	156	205.9	146.8	155.9	125.3
		118.9	161.7	190.3	144.7	150	121.3

		120.8	161.7	207.9	144.7	158.9	129.5
D2S2363		120.7	155.9	190.1(M)	134.3	156	125.2
D2S1379		122.6	155.9	203.8(F)	144.7	156	125.2
D2S1395		120.6	155.9	203.8	134.2	147.4	117.1
		122.6	155.9	205.8	134.2	156	125.2
		120.7	156	190.1	`144.7	151.8	121.2
		122.6	161.6	199.8	144.7	156	125.2
AFMA081WE1		120.7(M)	159.6	190.1(M)	144.7	151.6	121.2
D2S124		122.6(F)	159.6	192.1(F)	144.7	155.9	125.3
D2S2157		120.7	159.8	190.1	134.2	151.8	121.2
D2S1379		122.5	159.8	192.1	144.6	151.8	129.4
D2S1395		118.8	159.9	190.1	144.7	150	120.3
		120.7	161.9	190.1	146.8	155.5	125.3
D2S124		120.7	155.8(M)	205.8	134.2	151.7	121.2
D2S1379		122.5	161.4(F)	205.8	144.6	159.9	129.4
D2S1395		120.7	156.6	190.1	134.2	151.8	121.1
		122.5	161.6	205.8	144.6	155.9	125.2
		120.1	155.9	205.8	144.6	154.4	125.2
		122.1	159.9	207.8	144.6	158.9	129.3
AFMA081WE1		122.6(F)	159.7(F)	199.8(F)	134.2	146.3	116.1
D2S124		116.8(M)	155.9(M)	190.1(M)	144.7	155.9	125.2
D2S2363		118.7	159.7	190.1	134.3	155.9	125.1
D2S2157		122.6	161.6	199.8	146.8	155.9	125.1
		116.8	156.1	190.1	144.7	141.8	116.1
		120.6	160	190.1	144.7	159.9	116.1
D2S124		122.6	155.8(M)	190	138.2	151.7	117.6
D2S2157		122.6	159.6(F)	205.7	144.6	151.7	121.2
D2S1395		120.7	159.4	190	134.3	151.8	121.3

		122.6	159.4	205.7	144.7	155.9	125.4
		122.6	155.9	190.1	138.2	151.8	117.5
		122.6	163.5	205.8	149	151.8	121.2
D2S124		118.8	156.4(F)	190.1	144.7	151.8	121.3
D2S2363		120.7	161.5(M)	190.1	144.7	156	125.3
D2S1379		120.7?	154.3	190.1	144.7	147.4	117.1
D2S1395		120.7?	157.4	205.7	144.7	155.9	125.2
		122.5	157.8	190.2	134.3	151.8	122
		124.5	161.7	190.2	144.6	156	126.1
D2S2363		120.8	161.6	190.1(F)	144.7	151.8	121.2
D2S124		120.8	161.6	199.8(M)	146.8	156	125.3
AFMA081WE1		120.7	156	190.1	144.6	146.5	116.2
D2S2157		122.6	161.6	207.8	146.8	151.9	121.2
D2S1379		118.9	161.6	199.9	144.7	164.2	125.3
D2S1395		120.8	163.6	206	149	156.1	125.3
		118.7(F)	155.9	199.9(F)	138.3	151.9	121.3
D2S2363		120.6(M)	155.9	201.9(M)	144.7	156	125.3
AFMA081WE1		118.7	156	199.8	144.7	151.8	121.2
D2S2157		118.7	161.7	190.2	144.7	156	125.3
		120.7	156.1	201.9	138.3	156	125.4
		122.7	156.1	190.3	144.7	156	125.4
AFMA081WE1		120.6(F)	150.7(F)	190.2(F)	134.2	151.9	121.1
D2S124		118.6(M)	156.0(M)	205.8(M)	134.2	160.1	129.4
D2S2157		118.7	150.4	190.1	134.3	156	125.2
D2S1379		120.7	156	205.8	144.7	160.1	129.4
D2S1395		116.9	156	205.9	134.4	151.9	121.4
		118.8	159.9	205.9	144.7	156	125.3
D2S2363		120.6	157.7(F)	190.1(F)	144.6	155.9	125.3(M)

D2S2157		122.5	159.7(M)	205.7(M)	144.6	155.9	129.3(F)
D2S1395		120.6	157.8	190.2	144.7	156	125.3
		122.5	159.8	203.8	144.7	156	129.5
		120.7	159.5	192.1	144.8	156	117.1
		122.6	159.5	205.9	146.9	156	125.3
D2S2363		120.6	161.5	201.8(M)	144.6	147.3	117.1
D2S1379		122.6	161.5	205.8(F)	144.6	160	129.3
		120.6	159.8	190.1	144.7	147.4	117.2
		122.6	161.7	205.7	144.7	151.9	121.3
		120.7	156.1	192	134.3	156	125.4
		122.6	161.8	201.8	144.7	160.1	129.5
D2S2363		122.9	143.7	205.6(F)	133.4	115.5(F)	146.5(F)
D2S1395		122.9	143.7	207.6(M)	143.7	128.7(M)	160.5(M)
D2S1379		122.9	143.6	205.6	133.3	116.4	147.5
		122.9	143.6	205.6	143.6	116.4	147.5
		123	143.8	190	133.4	115.3	146.6
		123	143.8	207.7	143.8	128.5	160.4

Table S4: Summary of parental phenotypes

Epileptic phenotype	only father	only mother	both parents	neither parent	total	sperm collected	multiple samples collected
parental mosaic family	8	4	0	17	29	13	13
de novo family	5	4	1	73	83	46	2

Table S5: Primers for PASM detection

Primer	Forward	Reverse	Length
DS280-1	CATTGATTGGCCCTTTGGGG	AGAGAGGCCTATTTCTCTTGCAT	980
DS004-1	TGGGGAGATGAAAGTAGCATCA	AGTCGGGTGGCTTACTGTTG	826
DS001	AAGGCAGCAGAACGACTTGT	CCTTGGCATCACTCTGCTCA	386
DS003	CCATGAGCCTGAGACGGTTA	ACGCGGAACACAATCAGGAA	411
DS004-2	ACGCATGATTTCTTCACTGGT	CCAGCCAGCAGAGGTTGTAA	398
DS017	GAGGCGGTTCAAGCGCAG	TCAACTTTGAGACCTTTGGCAAC	402
DS035	CCAAAATGCATATCTTAAGTGGGT	TCATTTTCCAGCTGCGAGTT	398
DS094	TGCTAGCAATCCATCCCAGC	TTAACGCATGATTTCTTCACTGGT	390
DS101	AGTAGTAGTGATTGGCTGATAGGA	GGTGAACATGTACATCGCGG	397
DS125/DS128	AGTCTGTCAACATAACATAGTGGT	CTACTGTTTCTCTGCCCTCCT	398
DS136	TCTGGTCACTACTGACTATATCTGC	GGCGTAAATTTGTTTGCTGGC	398
DS152	GAATGCACTATTCCCAACTCACA	TGAACATTCCAGGCTAATGATACAA	400
DS164	TGAGGCTAATATGACAAAGATGCAA	GGGCACTTTAGAAATTGTGATTGTG	400
DS166	TCCAAAATGCATATCTTAAGTGGGT	CCAGCTGCGAGTTTTCAAGT	392
DS203	CCAAAATGCATATCTTAAGTGGGT	TCATTTTCCAGCTGCGAGTT	398
DS210	GAGGCGGTTCAAGCGCAG	TCAACTTTGAGACCTTTGGCAAC	402
DS276	GTTTTGAGCCAAGAAGAAATGGGA	AGGCAGCAGAACGACTTGTAAT	395
DS280-2	AAAAATTACATCCTTTACATCAAACT	TTTTGCATGCATAGATTTTCC	421
DS287	ATATAGCAGGCAGCAACGGC	TCCCCCTCTCTCCCATGTTT	378
DS296	TTGAGCCAAGAAGAAATGGGAG	AGAGTAAAAAGGCAGCAGAACG	401
DS306	TCCAAAATGCATATCTTAAGTGGGT	CCAGCTGCGAGTTTTCAAGT	392
DS307	AGTTGGCTGTTATCTTCAGTTTCT	GTGTCTAAAGTATCTTTGCACTGT	416
DS308	ATGGATTGTAATGGGGTGCTTCT	AGGACACAGTTTTAACCAGTTTGAT	399
DS312	TTCTCAAGGTTGCCGTTCTGT	GCTTTAGAGGGCGAGCAAAG	402
DS314	ACTTACAATGCTAATGGTTGTGTGG	TCATTGTGGGAAAATAGCATAAGCA	383
DS316	GCTTACTGTTGAGAATGGGTGC	GCATGATTTCTTCACTGGTTGGT	404

DS323	GTACTCATTTGGCAGAGAAAACACT	CATGAAAACATCCCTAAAGGCCAA	395
DS324	ACCTTCCCACACCTATAGAATCTT	AGGATGCACAAAGGAGTAGCTT	404
DS327	CCAAAATGCATATCTTAAGTGGGT	TCATTTTCCAGCTGCGAGTT	398
DS328	TTTTCCCAGAAGTCCTGAGTCAT	ATGAAATGGAGGTGTTGAAAATGC	395
DS329	TCTTAAAAGCATAAGCACTGATGGA	ACACTTTACGAAACAACTCTTTGTG	396

Table S6: BLAST and BLAT confirmation of similar genomic regions that would affect the detection of variants by mDDPCR and PASM.

Family	Sample	NA	MU	WT	mDDPCR Raindrop	mDDPCR lower	mDDPCR upper	Position	Exon	FMA PASM	PASM lower	PASM upper	Blast and Blat result (hg19)
DS164	Blood	7932075	60903	63008	49.15%	48.87%	49.43%	166915194	Exon2	50.0%	47.0%	53.0%	Unique
DS329	Blood	10226659	66460	66335	50.05%	49.78%	50.32%	166911262	Exon4	47.4%	45.1%	49.6%	Unique
DS307	Blood	10230824	52040	52698	49.69%	49.38%	49.99%	166905453	Exon7	46.1%	42.9%	49.4%	Unique
DS328	Blood	10329055	90285	89699	50.16%	49.93%	50.39%	166904273	Exon8	49.9%	47.5%	52.3%	Unique
DS001	Blood	7564829	31909	31417	50.39%	50.00%	50.78%	166904194	Exon8	49.2%	47.3%	51.1%	Unique
DS296	Blood	8253783	30905	30996	49.93%	49.53%	50.32%	166904178	Exon8	50.0%	48.9%	51.1%	Unique
DS276	Blood	8925070	32177	32578	49.69%	49.30%	50.08%	166904178	Exon8	49.7%	47.4%	52.0%	Unique
DS191	Blood	8395105	50841	134507	27.43%	27.23%	27.63%	166903460	Exon9	49.3%	48.1%	50.4%	2:166903440-166903480 and 2:166170418-166170452(SCN2A)
DS092	Blood	6260499	93827	287025	24.64%	24.50%	24.77%	166903392	Exon9	38.7%	37.6%	39.9%	2:166903372-166903412 and 2:166170486-166170526(SCN2A)
DS287	Blood	7605263	24583	24639	49.94%	49.50%	50.39%	166901753	Exon10	51.7%	51.5%	51.9%	Unique
DS312	Blood	6676979	32219	31519	50.55%	50.16%	50.94%	166900371	Exon11	43.9%	42.4%	45.3%	Unique
DS152	Blood	8974798	46238	46622	49.79%	49.47%	50.12%	166898844	Exon12	47.9%	45.8%	50.0%	Unique
DS314	Blood	8574182	40318	41130	49.50%	49.16%	49.85%	166895938	Exon14	41.4%	31.2%	52.1%	Unique
DS035	Blood	4129480	36597	36587	50.01%	49.64%	50.37%	166894440	Exon15	52.2%	50.9%	53.6%	Unique
DS203	Blood	8539326	32938	33381	49.67%	49.28%	50.05%	166894440	Exon15	53.5%	51.8%	55.3%	Unique
DS327	Blood	8228505	52078	52328	49.88%	49.58%	50.18%	166894436	Exon15	49.1%	47.1%	51.0%	Unique
DS166	Blood	6994465	28764	230040	11.11%	10.99%	11.24%	166894396	Exon15	52.4%	46.1%	58.6%	2:166894376-166894416 and 2:167133583-167133623(SCN9A) and 2:165986540-165986580(SCN3A) and 2:166201291-166201331(SCN2A)

DS306	Blood	7908998	69682	499213	12.25%	12.16%	12.33%	166894396	Exon15	50.7%	45.1%	56.3%	2:166894376-166894416 and 2:167133583-167133623(SCN9A) and 2:165986540-165986580(SCN3A) and 2:166201291-166201331(SCN2A)
DS120	Blood	4194203	355753	486622	42.23%	42.13%	42.34%	166894378	Exon15	32.8%	31.6%	34.0%	unique but with some highly homology sequences
DS324	Blood	9295167	50017	49540	50.24%	49.93%	50.55%	166892659	Exon16	60.3%	55.7%	64.9%	Unique
DS125	Blood	5800493	114211	114256	49.99%	49.78%	50.20%	166868765	Exon19	47.2%	44.6%	49.9%	Unique
DS128	Blood	4999049	31778	32598	49.36%	48.98%	49.75%	166868765	Exon19	49.2%	47.5%	51.0%	Unique
DS280	Blood	9232899	272138	275129	49.73%	49.59%	49.86%	166866246	Exon20	49.5%	48.9%	50.0%	Unique
DS136	Blood	6691798	12607	12758	49.70%	49.09%	50.32%	166859043	Exon21	46.9%	44.5%	49.2%	Unique
DS323	Blood	9620775	49228	49636	49.79%	49.48%	50.11%	166854686	Exon23	48.0%	46.0%	49.9%	Unique
DS308	Blood	8266044	21869	21981	49.87%	49.40%	50.34%	166852540	Exon24	52.9%	49.9%	55.9%	Unique
DS316	Blood	9108535	49909	50351	49.78%	49.47%	50.09%	166848864	Exon26	50.7%	49.4%	51.9%	unique but with some homology sequences
DS094	Blood	5424398	97801	530741	15.56%	15.47%	15.65%	166848852	Exon26	46.1%	42.8%	49.3%	2:166848832-166848872 and 2:166245199-166245239(SCN2A) and 2:165947755-165947795(SCN3A)
DS278	Blood	8112744	24983	176613	12.39%	12.25%	12.54%	166848800	Exon26	37.3%	36.6%	38.0%	2:166848780-166848820 and 2:166245251-166245291(SCN2A) and 2:167056222-167056262(SCN9A) and 2:165947703-165947743(SCN3A)
DS095	Blood	7524938	164210	1522767	9.73%	9.69%	9.78%	166848788	Exon26	40.3%	39.8%	40.8%	2:166848768-166848808 and 2:166245263-166245303(SCN2A) and 2:167056210-167056250(SCN9A) and 2:165947691-165947731(SCN3A) and 4:175842081-175842101(ADAM29)
DS004	Blood	4480577	48963	328352	12.98%	12.87%	13.08%	166848782	Exon26	56.1%	55.1%	56.4%	2:166848762-166848802 and 2:166245269-166245309(SCN2A)and 2:167056204-167056244(SCN9A) and 2:165947685-165947725(SCN3A)
DS210	Blood	6375796	89505	413729	17.79%	17.68%	17.89%	166848488	Exon26	48.6%	47.4%	49.8%	2:166848468-166848508 and 2:166245563-166245603(SCN2A) and 2:165947391-165947431(SCN3A)
DS134	Blood	5912931	174164	935895	15.69%	15.62%	15.76%	166848451	Exon26	32.3%	30.5%	34.2%	2:166848431-166848471 and 2:166245600-166245640(SCN2A) and 2:165947354-165947394(SCN3A)
DS093	Blood	5721658	233807	1264301	15.61%	15.55%	15.67%	166848439	Exon26	35.8%	34.1%	37.6%	
DS017	Blood	2960374	38050	194016	16.40%	16.25%	16.55%	166848438	Exon26	52.9%	39.4%	66.2%	
DS127	Blood	6715869	210421	1145698	15.52%	15.46%	15.58%	166848381	Exon26	38.2%	36.2%	40.1%	2:166848361-166848401 and 2:166245670-166245710(SCN2A) and 2:165947284-165947324(SCN3A)
DS101	Blood	1900277	8700	8491	50.61%	49.86%	51.36%	166848230	Exon26	46.2%	43.9%	48.4%	Unique

Table S7 : Versions of software

Software	Version
StepOne Software	2.1
Raindrop Analyst	2
R x64	3.2.2
Chromas	2.4.4
GeneMarker	2.2.0
Torrent Suite	4.4.2
Ion-analysis	4.4.8-1
Ion-chefupdates	4.4.5
Ion-dbreports	4.4.29-1
Ion-gpu	4.4.1-1
Ion-plpeline	4.4.11-1
Ion-plugins	4.4.14-1
Ion-torrentr	4.4.0-1
TS-Script	21.11.1
TS-LiveView	626
TS-DataCollect	487
TS-OS	20
TS-Graphics	36
yyxMosaicHunter	0.1.4
get_ref_alt_baseQ_corrected	0.9

Supplementary Web Resources

Burrows-Wheeler Aligner, http://bio-bwa.sourceforge.net/
BLAST, http://blast.ncbi.nlm.nih.gov/Blast.cgi
BLAT, http://genome.ucsc.edu/cgi-bin/hgBlat
Clinvar, www.ncbi.nlm.nih.gov/clinvar/
Exome Aggregate Consortium (ExAC) Browser, http://exac.broadinstitute.org/
iFish, http://ifish.cbi.pku.edu.cn/
Integrative Genomics Viewer (IGV), http://software.broadinstitute.org/software/igv/
MosaicHunter, http://mosaichunter.cbi.pku.edu.cn/
OMIM, http://www.omim.org

Supporting References:

1 Miller, S. A., Dykes, D. D. & Polesky, H. F. A simple salting out procedure for extracting DNA from human nucleated cells. *Nucleic acids research* **16**, 1215 (1988).

2 Gasteiger, E. *et al.* in *The proteomics protocols handbook* 571-607 (Springer, 2005).

3 White, H. E. *et al.* Accurate detection and quantitation of heteroplasmic mitochondrial point mutations by pyrosequencing. *Genet Test* **9**, 190-199, doi:10.1089/gte.2005.9.190 (2005).

4 Depienne, C. *et al.* Parental mosaicism can cause recurrent transmission of SCN1A mutations associated with severe myoclonic epilepsy of infancy. *Hum Mutat* **27**, 389-389 (2006).

5 Jones, A. C., Sampson, J. R. & Cheadle, J. P. Low level mosaicism detectable by DHPLC but not by direct sequencing. *Hum Mutat* **17**, 233-234 (2001).

6 Hiatt, J. B., Pritchard, C. C., Salipante, S. J., O'Roak, B. J. & Shendure, J. Single molecule molecular inversion probes for targeted, high-accuracy detection of low-frequency variation. *Genome Res* **23**, 843-854, doi:10.1101/gr.147686.112 (2013).

7 Lou, D. I. *et al.* High-throughput DNA sequencing errors are reduced by orders of magnitude using circle sequencing. *Proc Natl Acad Sci U S A* **110**, 19872-19877, doi:10.1073/pnas.1319590110 (2013).

8 Kennedy, S. R. *et al.* Detecting ultralow-frequency mutations by Duplex Sequencing. *Nature protocols* **9**, 2586-2606, doi:10.1038/nprot.2014.170 (2014).

9 Jee, J. *et al.* Rates and mechanisms of bacterial mutagenesis from maximum-depth sequencing. *Nature* **534**, 693-696, doi:10.1038/nature18313 (2016).

10 Kiss, M. M. *et al.* High-throughput quantitative polymerase chain reaction in picoliter droplets. *Anal Chem* **80**, 8975-8981 (2008).

11 Xu, X. *et al.* Early clinical features and diagnosis of Dravet syndrome in 138 Chinese patients with *SCN1A* mutations. *Brain & development* **36**, 676-681, doi:10.1016/j.braindev.2013.10.004 (2014).

12 Chen, W. W. *et al.* BEAMing and Droplet Digital PCR Analysis of Mutant IDH1 mRNA in Glioma Patient Serum and Cerebrospinal Fluid Extracellular Vesicles. *Molecular therapy. Nucleic acids* **2**, e109, doi:10.1038/mtna.2013.28 (2013).

13 Watanabe, M. *et al.* Ultra-Sensitive Detection of the Pretreatment EGFR T790M Mutation in

Non-Small Cell Lung Cancer Patients with an EGFR-Activating Mutation Using Droplet Digital PCR. *Clinical cancer research : an official journal of the American Association for Cancer Research* **21**, 3552-3560, doi:10.1158/1078-0432.CCR-14-2151 (2015).

14 Oxnard, G. R. *et al.* Noninvasive detection of response and resistance in EGFR-mutant lung cancer using quantitative next-generation genotyping of cell-free plasma DNA. *Clinical cancer research : an official journal of the American Association for Cancer Research* **20**, 1698-1705, doi:10.1158/1078-0432.CCR-13-2482 (2014).

15 Uchiyama, Y. *et al.* Ultra-sensitive droplet digital PCR for detecting a low-prevalence somatic GNAQ mutation in Sturge-Weber syndrome. *Scientific reports* **6**, 22985, doi:10.1038/srep22985 (2016).

16 Hindson, B. J. *et al.* High-throughput droplet digital PCR system for absolute quantitation of DNA copy number. *Anal Chem* **83**, 8604-8610, doi:10.1021/ac202028g (2011).

17 Xu, X. *et al.* Amplicon Resequencing Identified Parental Mosaicism for Approximately 10% of "de novo" SCN1A Mutations in Children with Dravet Syndrome. *Hum Mutat* **36**, 861-872, doi:10.1002/humu.22819 (2015).
